# Structural basis of iron piracy by human gut *Bacteroides*

**DOI:** 10.1073/pnas.2528036123

**Published:** 2026-05-01

**Authors:** Augustinas Silale, Yung Li Soo, Hannah Mark, Rachel N. Motz, Arnaud Baslé, Elizabeth M. Nolan, Bert van den Berg

**Affiliations:** ^a^Biosciences Institute, Newcastle University, Faculty of Medical Sciences, Newcastle upon Tyne NE2 4HH, United Kingdom; ^b^Department of Chemistry, Massachusetts Institute of Technology, Cambridge, MA 02139

**Keywords:** iron piracy, xenosiderophores, gut microbiota, TonB-dependent transporter, lipoprotein

## Abstract

Iron is an essential element that can be growth-limiting in microbial communities, particularly those present within host organisms. To acquire iron, many bacteria secrete siderophores, secondary metabolites that chelate ferric iron. These iron chelates can be transported back into the cell via TonB-dependent transporters in the outer membrane, followed by intracellular liberation of the iron. Pathogenic *Escherichia coli* and *Salmonella* produce siderophores during gut infection. In response to iron starvation, the human gut symbiont **Bacteroides* thetaiotaomicron* upregulates an iron piracy system, XusABC, which steals iron-bound siderophores from the invading pathogens. Here, we investigated the molecular details of xenosiderophore uptake across the outer membrane by the XusAB complex. Our crystal and cryogenic electron microscopy structures explain how the XusB lipoprotein recognizes iron-bound xenosiderophores and passes them on to the XusA TonB-dependent transporter. Moreover, we show that Xus homologues can transport a variety of siderophores with different iron-chelating functional groups.

## Significance

Iron is an essential micronutrient, and its low bioavailability can be growth-limiting in microbial communities. Bacteria have evolved mechanisms to steal iron from their competitors, resulting in complex interactions within microbial ecosystems, including the human gut. Here, we investigate how gut commensal *Bacteroides* spp. use cell surface lipoproteins and TonB-dependent transporters to steal iron-chelating small molecules, siderophores, produced by pathogenic bacteria and fungi. We illuminate the mechanistic details of a strategy that allows commensal bacteria to thrive under iron starvation during infection and avoid being outcompeted by invading pathogens.

Iron is essential for most organisms, including almost all bacteria. It is a protein cofactor required for enzymatic reactions and electron transport during various cellular processes. While iron is one of the most abundant transition metals on Earth, its bioavailability is limited due to the extremely low solubility (~10^−18^ M) of its predominant ferric (Fe^3+^) form at physiological pH (1). Many Gram-negative bacteria release siderophores, iron-chelating secondary metabolites, into the environment which, once bound to ferric iron, can be transported back into the cell via TonB-dependent transporters in the outer membrane (OM) (2). A classic example is enterobactin (Ent), which is produced by Enterobacteriaceae and consists of three iron-chelating catecholate groups connected via amide linkers to a cyclic triserine lactone (3, 4). Ferric enterobactin (FeEnt) is taken up by the FepA and IroN transporters in *Escherichia coli* and *Salmonella* (5–7), and iron is liberated from the FeEnt complex inside the cell (8, 9). Siderophore-mediated iron scavenging is important for pathogens during infection, when the host restricts iron availability to starve invading bacteria via nutritional immunity (10–12). Additionally, secreted siderophores are not necessarily taken up by the same bacterium that produced them, resulting in complex iron availability-dependent interactions in microbial communities (13).

**Fig. 1.**
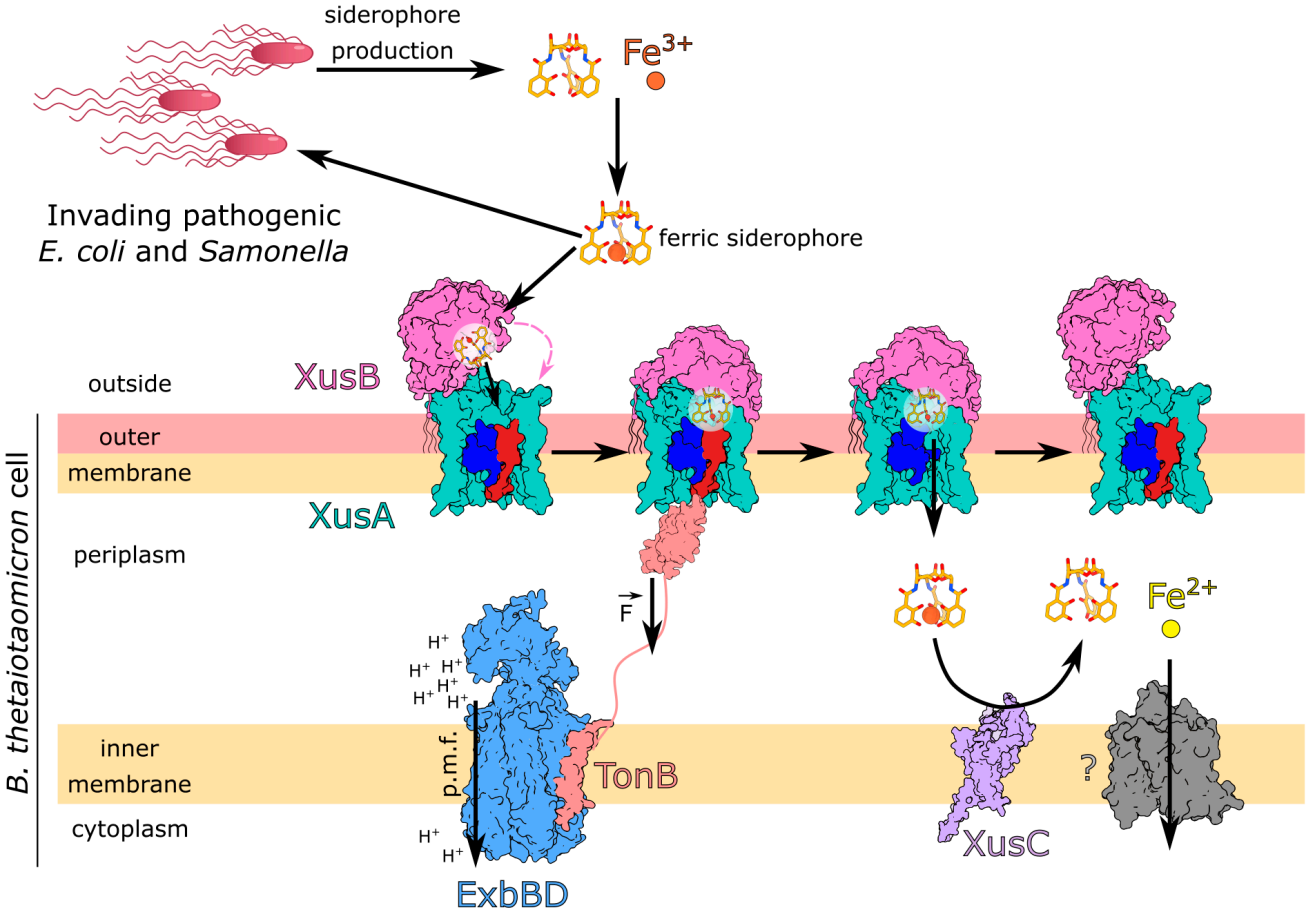
*B. theta* xenosiderophore utilization system model. Siderophores produced by pathogens (xenosiderophores) chelate ferric iron, and the ferric siderophore complex is bound by the surface-exposed lipoprotein XusB. The ferric siderophore is transferred from XusB to its partner protein XusA, a TonB-dependent transporter. Ligand binding causes conformational changes throughout XusA, resulting in exposure of a short N-terminal motif, the TonB box, which is recognized by the C-terminal domain of the inner membrane TonB protein. The ExbBD complex transduces energy stored in the proton motive force (p.m.f.) via TonB into a mechanical pulling force exerted on the plug domain of XusA (blue and red). The mechanically labile portion of the plug (red) is displaced from the XusA barrel lumen and a transport channel for the ferric siderophore complex is opened. After the ligand diffuses across the outer membrane, the inner membrane iron–siderophore reductase XusC reduces ferric iron to its ferrous form, liberating the ion. Ferrous iron is then likely transported by an unknown inner membrane protein into the cytoplasm.

The gastrointestinal tract is home to a diverse community of microorganisms that cooperate and compete for available nutrients both with the host and between themselves (14, 15). Depriving the gut microbiota of iron eventually results in irreversible structural changes in gut microbial communities (16, 17). Bacteroidota, the dominant phylum of diderm bacteria in the distal gut, are not known to produce siderophores. **Bacteroides* thetaiotaomicron* has been shown to prefer heme as an iron source over soluble ferrous iron (18). *B. theta* likely acquires heme from the diet (19) as well as from dead intestinal cells and dead bacteria. Iron-bound siderophores produced by commensal bacteria and fungi as well as various pathogens are another source of bioavailable iron for gut bacteria that have transporters to take them up (20). It was recently shown that during *Salmonella* infection in the mouse gut, when iron availability becomes limiting, *B. theta* upregulates a novel iron uptake system that steals iron-bound siderophores produced by the pathogenic *Salmonella* (21). This xenosiderophore (i.e., foreign siderophore) utilization system (Xus) consists of the TonB-dependent transporter XusA (BT_2065), the surface-exposed lipoprotein XusB (BT_2064) and the PepSY domain-containing inner membrane protein XusC (BT_2063) (21, 22) (Fig. 1). The XusABC system provides resilience to *B. theta* during infection, but XusB secreted by *B. theta* and bound to xenosiderophores also acts as an iron reservoir for the pathogen (21, 22). It is therefore important to understand the molecular details of this iron piracy system to obtain a deeper understanding of pathogen–symbiont interactions inside the gut.

Here, we present crystal structures of the apo- and xenosiderophore-bound XusB lipoproteins from several Bacteroidota species, which reveal the mechanism of xenosiderophore capture. We also use single particle cryo-EM to investigate how XusB interacts with the XusA TonB-dependent transporter. In vitro binding studies and structural bioinformatics analyses reveal distinct subclasses of xenosiderophore utilization systems in Bacteroidota. Together, our results provide mechanistic insights into xenosiderophore uptake across the OM of the gut commensal *B. theta* and the pathobiont *Bacteroides fragilis*.

## Results

### Crystal Structures of Apo and Xenosiderophore-Bound *B. theta* XusB.

**Fig. 2.**
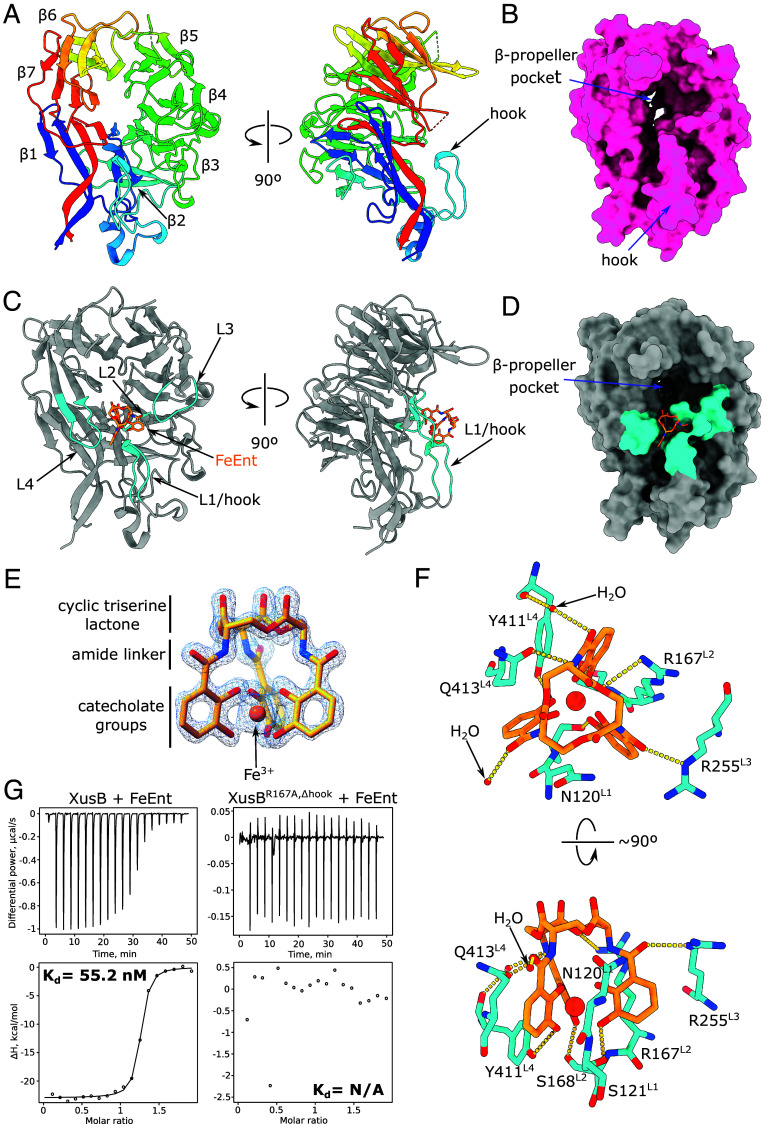
BtXusB binding to FeEnt. (*A*) Crystal structure of apo BtXusB at 1.56 Å. The cartoon is colored in rainbow; the N terminus is blue, the C-terminus is red. Blades of the β-propeller (β1-7) are labeled starting from the N terminus. (*B*) Surface representation of apo BtXusB. (*C*) Cocrystal structure of BtXusB bound to FeEnt at 1.5 Å. FeEnt is depicted as an orange stick model. The four FeEnt binding loops (L1–L4) are shown in cyan. (*D*) Surface representation of BtXusB bound to FeEnt. (*E*) FeEnt model fit into the 2mF_o_-DF_c_ electron density map at 2σ. (*F*) BtXusB residues interacting with FeEnt. The yellow dashed lines show likely hydrogen bonds. (*G*) Representative ITC experiments where 250 μM FeEnt was titrated into 25 μM BtXusB (n = 4 experiments) or 25 μM BtXusB^R167A,Δhook^ variant (n = 2 experiments). Integrated heats were fitted to a single binding site model, giving the apparent K_d_ value for the XusB-FeEnt titration. No K_d_ value could be determined for the XusB^R167A,Δhook^ titration.

We expressed recombinant XusB of *B. theta* (BtXusB; UniProt accession Q8A622) lacking the signal sequence and the lipid anchor cysteine in *E. coli* BL21(DE3) and determined its crystal structure using data to 1.56 Å resolution (Fig. 2 *A* and *B* and *SI Appendix*, Table S1). BtXusB has a seven-bladed β-propeller fold with a distinct 15-residue loop, which we termed the hook, inserted into the β2 blade and protruding outward from the β-propeller (Fig. 2*A*). The β-propeller pocket (Fig. 2*B*) has been suggested to bind FeEnt based on recent computational docking (22).

We cocrystallized recombinant BtXusB with FeEnt and determined the crystal structure of the complex to 1.50 Å. BtXusB binds FeEnt via four loops (Fig. 2*C*), including the hook. Interestingly, FeEnt binds off the β-propeller pocket rather than in it, and the distance to the previously modeled binding site is ~10 Å (Fig. 2*D*) (22), underscoring the importance and value of experimental protein–ligand structure determination. The FeEnt electron density in the cocrystal structure is of very high quality (Fig. 2*E*), with the cyclic triserine lactone, amide linkers, and Fe^3+^-ligating catecholate groups fully resolved. The catecholate arms of FeEnt are not perpendicular to the triserine lactone ring plane but slanted to one side (Fig. 2*E* and Movie S1), in agreement with the structure of FeEnt in isolation (4). FeEnt does not undergo conformational changes upon binding to BtXusB. Instead, BtXusB loops close in on FeEnt, forming a 419 Å^2^ interaction interface (*SI Appendix*, Figs. S1 and S2 and Movie S2). All three catecholate arms of FeEnt interact with BtXusB loops L1-4 (Fig. 2 *C* and *F*). Side chains of N120, R167, and Q413 slot between the slanted catecholate arms, while S121, S168, and Y411 side chains form hydrogen bonds with the *meta* oxygens of the catecholate groups (Fig. 2*F* and Movie S1). One amide linker of FeEnt hydrogen-bonds to a single water molecule, another to a water molecule and the side chain of Q413, and the third to the side chain of R255 (Fig. 2*F*). The side chain of R167 forms a hydrogen bond with the triserine lactone ring—the only interaction between BtXusB and this part of FeEnt.

BtXusB binds FeEnt with a dissociation constant value of ~55 nM as determined by isothermal titration calorimetry (ITC) (Fig. 2*G*). We constructed a BtXusB variant, BtXusB^R167A,Δhook^, with the R167A substitution and deletion of residues 119 to 122 which form the tip of the hook. Titration of FeEnt into BtXusB^R167A,Δhook^ resulted in reduced injection heats and no saturation (Fig. 2*G*), which we interpret as lack of binding. The ITC results strongly suggest that the binding site observed in the cocrystal structure is the only FeEnt binding site on BtXusB. The dissociation constant of BtXusB and FeEnt is comparable to reported values for other TonB-dependent transporter-associated proteins involved in iron uptake: ~126 nM for the TbpB lipoprotein from *Neisseria* which binds human transferrin (23), and ~2 nM for secreted *Serratia marcescens* HasA which binds heme (24).

**Fig. 3.**
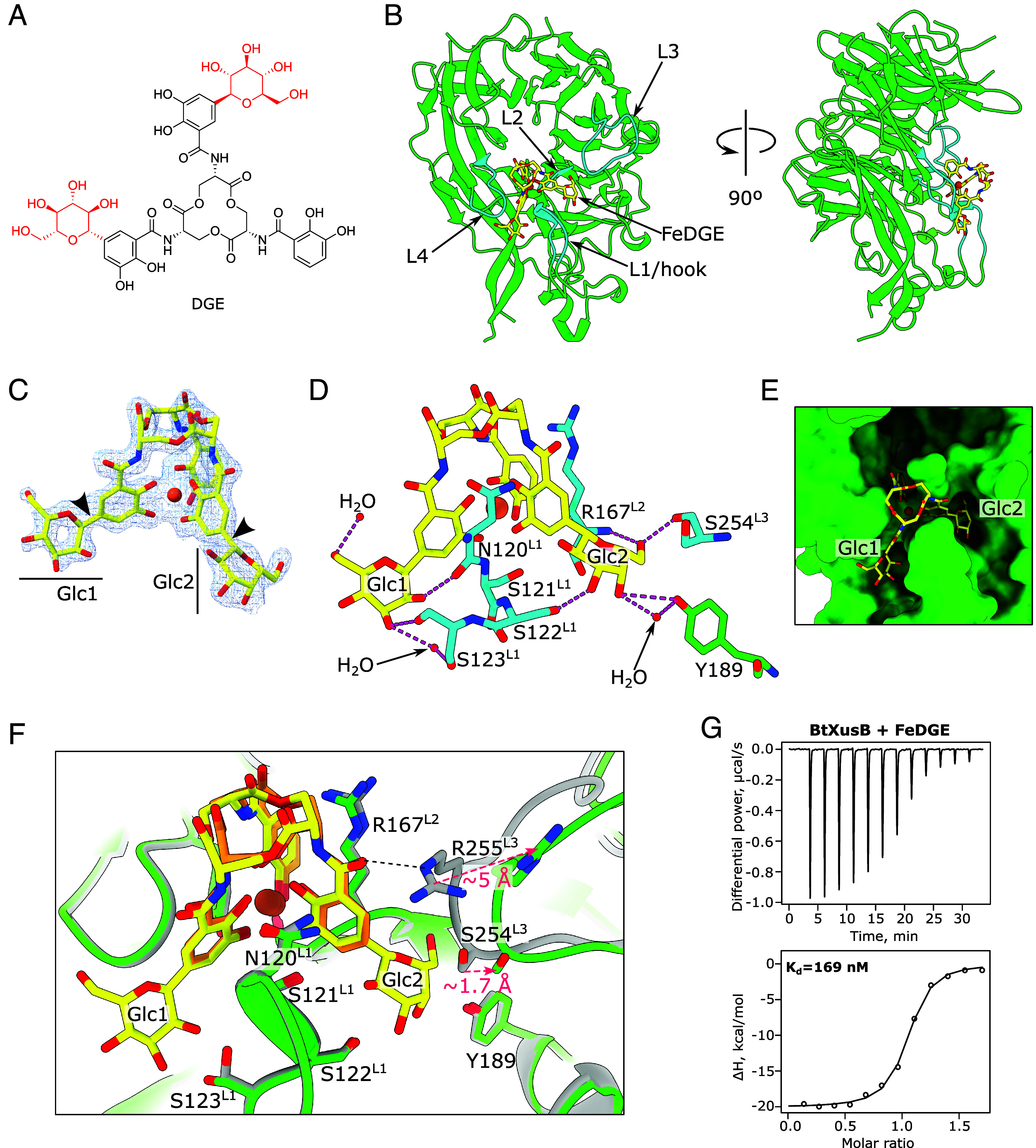
BtXusB binding to FeDGE. (*A*) Chemical structure of DGE. The glucosyl groups are in red. (*B*) Crystal structure of BtXusB (green) bound to FeDGE (yellow). The siderophore-binding loops are shown in cyan. (*C*) FeDGE model fit into the 2mF_o_-DF_c_ electron density map at 1σ. The arrowheads point to the C–C bonds between the catechol groups and the C1 atoms of the glucosyl moieties. (*D*) Hydrogen bonding network formed between the glucosyl groups of FeDGE and BtXusB. (*E*) The Glc1 and Glc2 groups of FeDGE occupy slots between the xenosiderophore-binding loops of BtXusB (green surface). (*F*) Superposition of BtXusB–FeEnt (gray and orange, respectively) and BtXusB–FeDGE (green and yellow, respectively) crystal structures. Hook loop residues N120-S123 and L2 R167 are in almost identical conformations in the two structures, but L3 is pushed away in the FeDGE structure due to a clash between Glc2 of FeDGE and S254 of L3. The black dashed line indicates the hydrogen bond between R255 and the amide carbonyl of FeEnt. (*G*) Representative ITC experiment where 154 μM FeDGE was titrated into 17.3 μM BtXusB (n = 2 experiments). Integrated heats were fitted to a single binding site model, giving the apparent K_d_ value.

Ent secreted by pathogens and commensals can be taken up by any other Gram-negative bacterium that expresses suitable TonB-dependent transporters, such as FepA and XusA (5, 21). Furthermore, as part of the innate immune response host cells secrete lipocalin-2, which sequesters FeEnt and deprives pathogens of iron (25, 26). Therefore, production of Ent by the pathogen can be less effective during infection. *Salmonella* and some *E. coli*, e.g. many uropathogenic strains (27), have in turn evolved a strategy to prevent waste of resources and secure iron by making chemically modified versions of Ent that require the TonB-dependent transporter IroN for import and do not bind to lipocalin-2, such as di-*C*-glucosylenterobactin (DGE), also known as salmochelin S4 (28, 29). DGE has the same core structure as Ent, with two of the catechol groups *C-*glucosylated at the C5 position (Fig. 3*A*). Notably, *E. coli* FepA does not import DGE (7). We soaked apo BtXusB crystals with iron-bound DGE (FeDGE) and determined the crystal structure of FeDGE-bound BtXusB to 1.80 Å resolution (Fig. 3*B*). One of two protein chains in the asymmetric unit had FeDGE bound, which we could confidently build into the ligand density (Fig. 3 *B* and *C*). BtXusB binds FeDGE via the same loops as FeEnt with additional interactions with the two glucosyl modifications, Glc1 and Glc2 (Fig. 3*D*). Glc1 hydroxyl groups hydrogen-bond to two water molecules and the hook loop via the backbone carbonyl oxygen of N120 and the side chain of S123. Glc2 contacts the hook via the side chain of S122, L2 via the backbone of R167, and L3 via the backbone of S254. Additionally, Glc2 interacts with a single water molecule and the hydroxyl group of Y189, which is not part of the four loops that bind FeEnt. The electron density for Glc1 was weaker than for Glc2 (Fig. 3*C*), which suggests that Glc1 is bound less tightly than Glc2. The two glucosyl modifications slot between the ligand-binding loops (Fig. 3*E*). One notable difference between the FeEnt- and FeDGE-bound structures is that the amide linker of one catechol arm of FeDGE does not hydrogen-bond with the side chain of L3 R255 as observed for FeEnt (Fig. 3*F*). This rearrangement is likely the result of the C6 atom of Glc2 nudging the side chain of S254 by ~1.7 Å and pushing the entire L3 further away from the siderophore. Consequently, the side chain of R255 swings away by ~5 Å and can no longer contact the amide linker. ITC data suggest that the amide–R255 interaction is important for tight xenosiderophore binding, as the FeDGE–BtXusB interaction has a two to threefold lower apparent dissociation constant value compared to FeEnt (Fig. 3*G* and *SI Appendix*, Table S2), even though the glucosyl modifications of FeDGE form additional contacts with BtXusB. In addition, the decreased mobility of the constrained Glc modifications likely results in an entropic penalty for bound FeDGE. ITC data fitting results support this prediction, as the BtXusB-FeDGE interaction has an estimated –TΔS value ~2.3 kcal/mol smaller than the BtXusB–FeEnt interaction, while the ΔG terms are similar for both with a difference of ~0.6 kcal/mol (*SI Appendix*, Table S2).

### Cryo-EM Structure of Native XusAB Complex From *B. theta*.

**Fig. 4.**
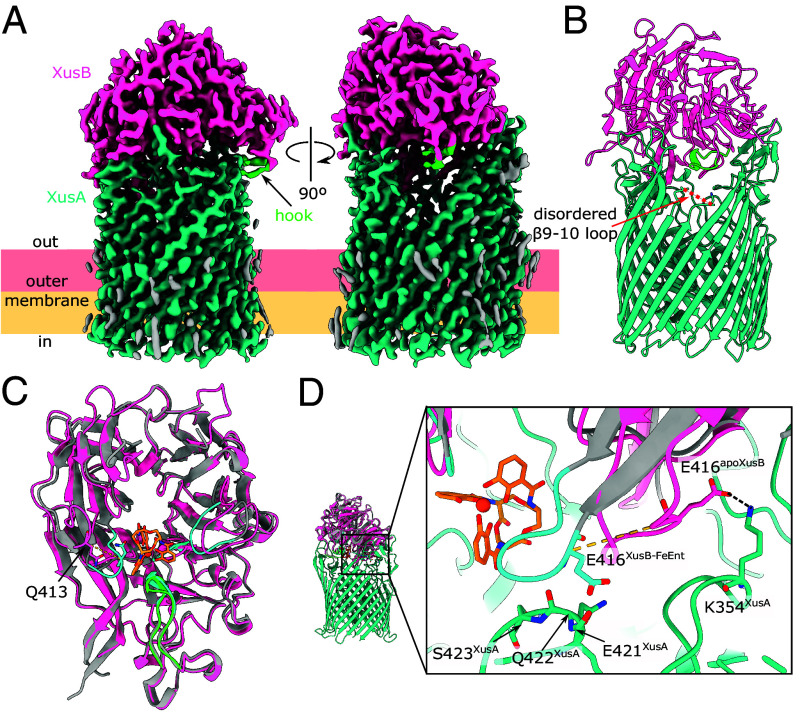
Cryo-EM structure of the native XusAB complex from *B. theta*. (*A*) Single particle cryo-EM reconstruction of the XusAB complex at 2.7 Å global resolution. (*B*) Model built into the cryo-EM density in cartoon representation. The red dashed line depicts the disordered extracellular loop between β strands 9 and 10 of the XusA β-barrel, corresponding to residues 464-473. (*C*) Structural alignment of the apo-XusB structure observed in the cryo-EM reconstruction (hot pink, hook in green) and the XusB-FeEnt cocrystal structure (gray, FeEnt binding loops in cyan). Cα-Cα RMSD = 1.6 Å. The dashed yellow line corresponds to a distance of 7.7 Å between the Cα atoms of Q413, which is part of the L4 FeEnt binding loop, observed in the two structures. (*D*) Close-up view of XusB L4 in the apo cryo-EM structure and the XusB-FeEnt cocrystal structure, viewed from a different orientation compared to (*C*). Coloring as in (*C*), XusA is in light sea green. The yellow dashes correspond to a distance of 11.3 Å between the Cα atoms of E416 in the two structures. The black dashes show a salt bridge between K354 of XusA and E416 of XusB in the cryo-EM structure. Residues E421, Q422, and S423 are part of the β7-8 extracellular loop of XusA.

FeEnt is sufficient to rescue growth of *B. theta* under iron limiting conditions (*SI Appendix*, Fig. S3). We reasoned that the proteins encoded by the Xus operon would be expressed to high enough copy number for purification and structural characterization if the cells were cultured under iron-limiting conditions. Whole-cell western blots of a *B. theta bt2064-his* strain grown in minimal medium supplemented with the iron chelator bathophenanthroline disulfonate (BPS) showed that iron starvation is sufficient to induce XusB expression (*SI Appendix*, Fig. S3 and *SI Methods*). This observation suggests that there is no mechanism for sensing xenosiderophores and that Xus is upregulated solely in response to iron starvation. We purified the native XusAB complex from the *B. theta bt2064-his* strain (*SI Appendix*, Fig. S3 and *Materials and Methods*). We determined the structure of XusAB by single particle cryo-EM to a global resolution of 2.7 Å, with local resolution extending to 2.2 Å (Fig. 4*A* and *SI Appendix*, Figs. S4 and S5 and Table S3). The resolved portion of XusA has a classic TonB-dependent transporter fold: a micelle-embedded 22-strand β-barrel occluded by an N-terminal plug domain (2). The XusA extreme N-terminal carboxypeptidase-like domain of unknown function was not resolved in our structure likely because it is connected to the barrel via a flexible linker. The conformation of XusB in the XusAB complex is almost identical to that observed in the apo XusB crystal structure, with Cα-Cα RMSD = 0.9 Å. We did not observe any alternative conformations, subcomplexes, or movement of XusA and XusB in the cryo-EM data.

XusB sits on top of the extracellular side of XusA like a lid, reminiscent of *B. theta* TonB-dependent transporter and surface-exposed lipoprotein complexes that take up glycans and vitamin B_12_ (30, 31). The interaction interface of XusA and XusB is extensive: PISA analysis indicates 52 hydrogen bonds and 6 salt bridges, with a total interaction surface area of 2,795 Å^2^ (*SI Appendix*, Fig. S6). XusB interacts with every extracellular loop of XusA, except for the loop between barrel strands β9 and β10, which is disordered (Fig. 4*B*). Although we did not observe FeEnt in the cryo-EM structure, the XusB hook facing toward a cavity enclosed by the XusAB complex (Fig. 4 *A* and *B*) indicates that FeEnt binds inside this cavity. We speculate that XusB moves in a hinge-like motion opening and closing the XusAB cavity like a lid, thus transiently allowing extracellular xenosiderophores access to their binding site on the inward-facing side of XusB.

XusB binds FeEnt with high affinity (Fig. 2*G*), but FeEnt must somehow be transferred from XusB to XusA to achieve transport across the OM. Structural alignment of the cryo-EM XusB structure and the XusB-FeEnt cocrystal structure reveals that Q413, part of L4, must undergo a shift of 7.7 Å to interact with FeEnt (Fig. 4*C*). However, the position of L4 in the XusB-FeEnt structure would result in clashes with the XusA β7-8 extracellular loop within the apo complex (Fig. 4*D*). Furthermore, L4 in the apo complex is stabilized via a salt bridge between E416 and K354 of XusA β5-6 extracellular loop. This suggests that the FeEnt-bound state of XusB when in complex with XusA might be short-lived despite the high affinity of XusB for FeEnt. The XusA β7-8 extracellular loop could displace the L4 of XusB, aided by formation of the E416-K354 salt bridge, thus disrupting the FeEnt binding pocket and releasing the xenosiderophore to diffuse toward XusA, which would transport it across the OM. We envisage that the transfer of FeDGE from XusB to XusA proceeds via an identical mechanism to FeEnt.

We observed unexplained density extending from the side chain of T401, which is part of the XusA β7-8 extracellular loop (*SI Appendix*, Fig. S7). Together with flanking residues this threonine forms a DTA sequence, which matches the Bacteroidota O-glycosylation motif (32). We therefore conclude that the cryo-EM density extending from T401 corresponds to a glycan chain. Four sugar units can be discerned, including the branching deoxyhexose previously identified in the *B. fragilis* O-glycan (33). The T401 glycosylation site is located near residues 421-423 which are implicated in FeEnt release from XusB (Fig. 4*D* and *SI Appendix*, Fig. S7). The side chain of T401 and the O-glycan face the solvent rather than XusB, but we cannot rule out that the glycan modification affects the conformation of neighboring XusA extracellular loops and that it could be functionally important.

### Structural Variety of XusB Homologues.

**Fig. 5.**
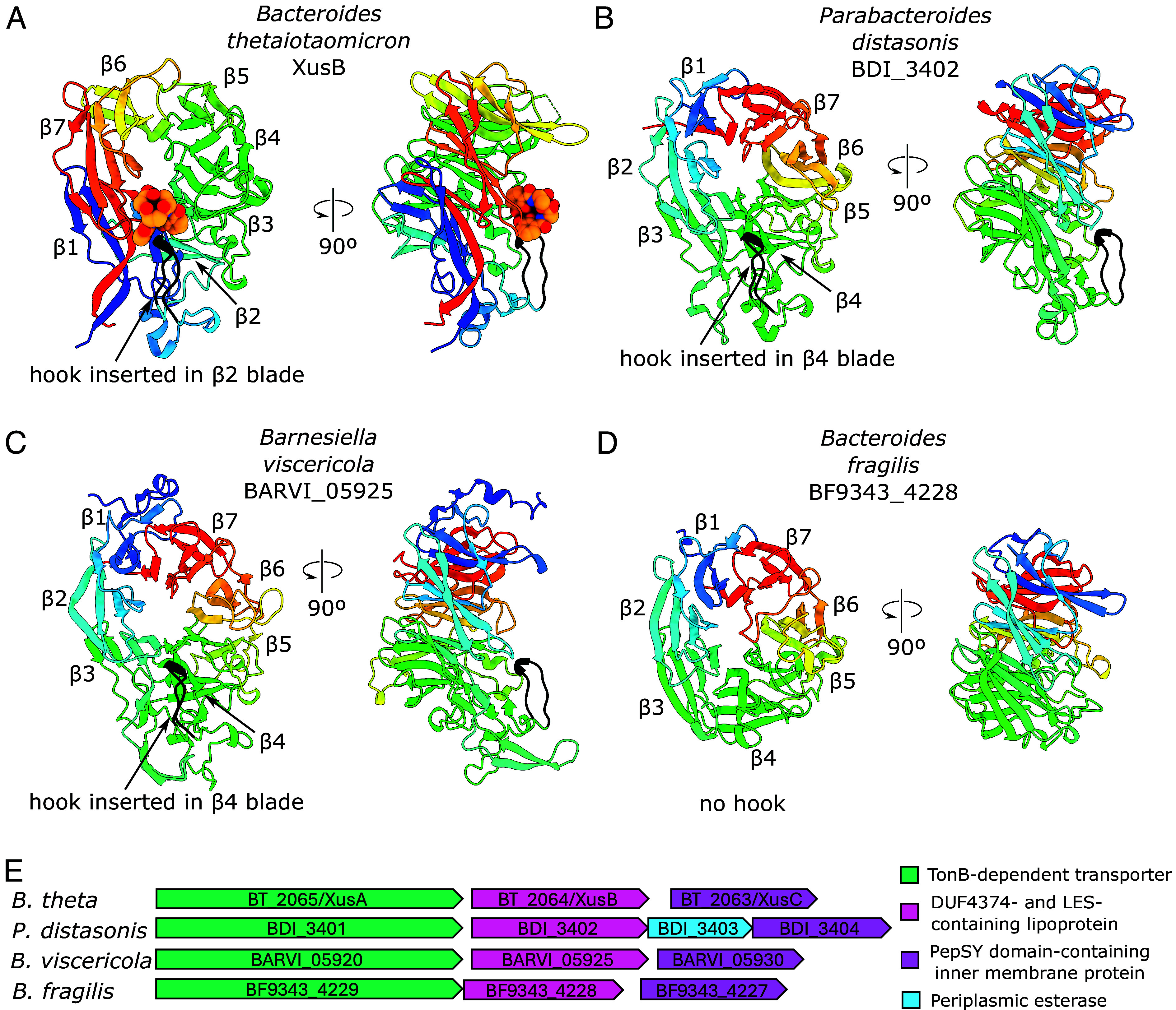
Structural variety of DUF4374-containing proteins. (*A*) BtXusB–FeEnt cocrystal structure. FeEnt atoms are displayed as spheres. (*B*) *P. distasonis* DSM 20701 BDI_3402 (59.52% identity to *B. theta* XusB), (*C*) *B. viscericola* DSM 18177 BARVI_05925 (43.36% identity to BtXusB), and (*D*) *B. fragilis* NCTC 9343 BF9343_4228 (20.21% identity to BtXusB) AlphaFold2 (37) models. Views were generated from a superposition, β-propeller blades are labeled β1-7 starting from the N terminus. All models are colored in rainbow, from the N terminus in blue to the C-terminus in red; the hook is in black. AlphaFold2 model prediction confidence is shown in *SI Appendix*, Fig. S9. (*E*) Genetic context of the selected XusB homologues. DUF, domain of unknown function; LES, lipoprotein export signal (35).

BtXusB is annotated as a DUF4374-containing protein in UniProt (34) and has a β-propeller fold as demonstrated by our structural data. We investigated the predicted structures of DUF4374-containing homologues. The top hits from protein BLAST searches of the BtXusB sequence against Bacteroidota genomes have a sequence identity of around 60%. The reason for the relatively low sequence identity for the top BLAST hits is that the BtXusB hook position inside the β2 propeller blade is unique (Fig. 5*A*). Other Bacteroidota XusB homologues, such as *Parabacteroides distasonis* BDI_3402 (59.5% identity; UniProt accession A6LHE0) and *Barnesiella viscericola* BARVI_05925 (43.4% identity; UniProt accession W0ET74), are predicted to have their hook inserted in the β4 blade or, in the case of *B. fragilis* BF9343_4228 (20.2% identity; UniProt accession Q5L7E3), do not appear to have a hook at all (Fig. 5 *B*–*D* and *SI Appendix*, Fig. S8). These DUF4374-containing proteins are likely part of genuine xenosiderophore utilization systems, rather than performing some other function. They all contain a lipoprotein export signal that directs proteins to the outer leaflet of the OM (35). Additionally, they are encoded next to TonB-dependent transporters and PepSY domain-containing inner membrane proteins that likely reduce siderophore-bound ferric iron to facilitate dissociation of the iron–siderophore complex (36) (Fig. 5*E*). *P. distasonis* also encodes a periplasmic esterase (BDI_3403) in the same operon which might liberate siderophore-bound iron via siderophore hydrolysis, as shown for FeEnt in *E. coli* (8, 9).

Conservation of the hook and L2 amino acid sequences between the *B. theta, P. distasonis* and *B. viscericola* homologues (*SI Appendix*, Fig. S8) suggests that, despite differences in position of the hook within the β-propeller, *P. distasonis* and *B. viscericola* homologues might still bind FeEnt, FeDGE, and perhaps other catecholate siderophores. However, the *B. fragilis* homologue is unlikely to bind FeEnt due to lack of all FeEnt-binding regions observed in the BtXusB–FeEnt cocrystal structure, consistent with a previous report that *B. fragilis* cannot utilize catecholate xenosiderophores (38).

The amino acid sequence length of BtXusB BLAST hits follows a bimodal distribution with peaks at 410 and 465 amino acids (*SI Appendix*, Fig. S10). *B. theta, P. distasonis* and *B. viscericola* homologues (464, 471, and 491 residues, respectively) belong to the longer group, while the *B. fragilis* homologue (406 residues) belongs to the shorter group. Together with the differences in the xenosiderophore-binding loop regions, the bimodal distribution suggests there are at least two different subtypes of DUF4373-containing proteins.

### *B. viscericola* XusB Binds Ferric Enterobactin.

We produced recombinant XusB from *B. viscericola* (BvXusB) to investigate its xenosiderophore-binding properties. We observed binding of FeEnt to BvXusB in ITC with similar affinity to BtXusB (*SI Appendix*, Fig. S11*A*). We attempted to investigate the molecular details of the interaction between BvXusB and FeEnt and obtained the crystal structure of apo BvXusB (*SI Appendix*, Fig. S11*B* and Table S1), which confirmed the computational prediction. However, we could not cocrystallize BvXusB with FeEnt, and BvXusB crystals soaked with FeEnt did not diffract. Superposition of the BtXusB–FeEnt and apo BvXusB structures suggests that most BtXusB residues involved in FeEnt binding are present in BvXusB, even though the hook loop in BvXusB is inserted in the β4 blade rather than in the β2 blade as in BtXusB (*SI Appendix*, Fig. S11*C*). Based on these structural similarities and AlphaFold 3 predictions of BvXusB in complex with FeEnt and FeDGE (*SI Appendix*, Fig. S12), we expect that BvXusB and other XusB homologues with the hook inserted in the β4 blade interact with FeEnt and FeDGE in a very similar manner to BtXusB.

### *B. fragilis* XusB Binds Ferrichrome.

**Fig. 6.**
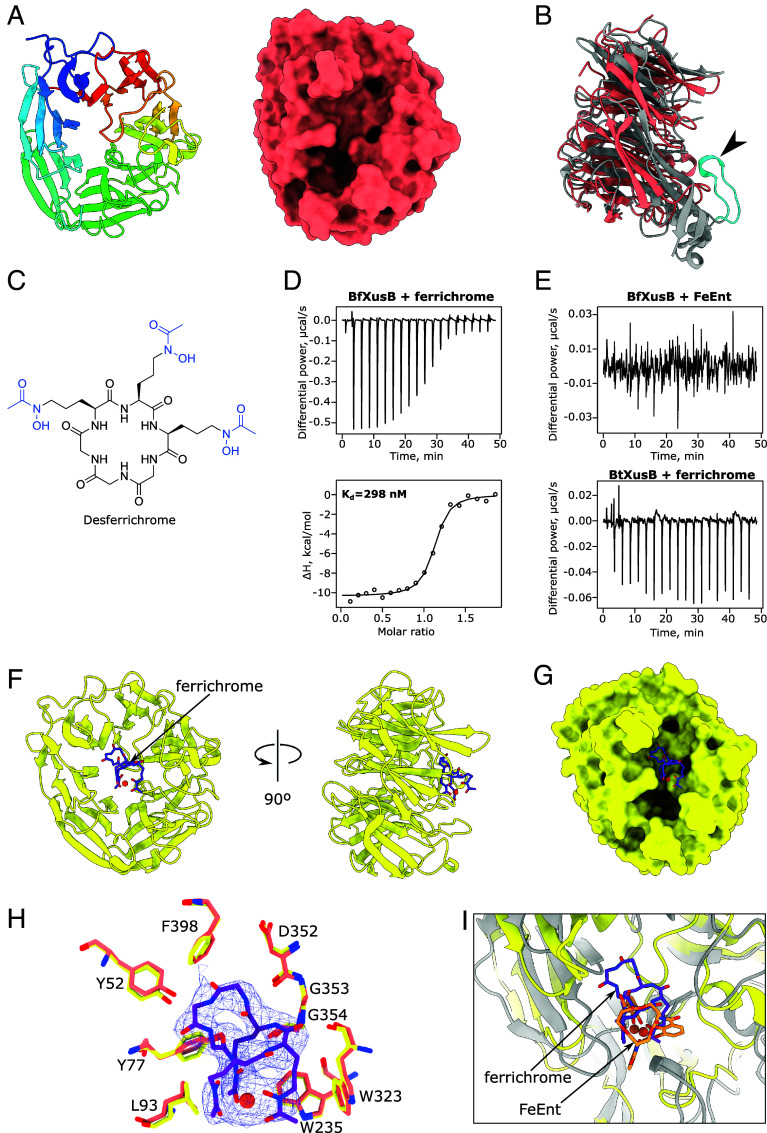
*B. fragilis* XusB binds ferrichrome. (*A*) Crystal structure of apo BfXusB at 1.77 Å shown in cartoon (*Left*) and surface (*Right*) representations. (*B*) Superposition of apo BfXusB and apo BtXusB crystal structures generated using Matchmaker in ChimeraX (Cα-Cα RMSD = 1.2 Å between 140 pruned atom pairs; 3.8 Å across all 249 pairs). BtXusB is in gray; the hook is highlighted in cyan and indicated by the arrowhead. (*C*) Chemical structure of desferrichrome. The ferric iron-ligating hydroxamate groups are colored in blue. (*D*) Representative ITC experiment where 250 μM ferrichrome was titrated into 25 μM BfXusB (n = 4 repeats). Integrated heats were fitted to a single binding site model, giving the apparent K_d_ value. (*E*) Representative ITC experiments where 250 μM FeEnt was titrated into 25 μM BfXusB (n = 2 experiments) and 250 μM ferrichrome was titrated into 25 μM BtXusB (n = 2 experiments). (*F*) Crystal structure of ferrichrome-bound BfXusB to 3.32 Å. (*G*) Surface representation of BfXusB with ferrichrome shown as a stick model inside the shallow β-propeller pocket. (*H*) Ferrichrome model fit into the 2mF_o_-DF_c_ electron density map at 1σ. Residues that likely interact with ferrichrome are shown as yellow stick models. The same residues in the apo BfXusB crystal structure are shown as salmon stick models. (*I*) Superposition of BfXusB-ferrichrome (yellow and purple) and BtXusB–FeEnt (gray and orange) crystal structures (Cα-Cα RMSD between 140 pruned atom pairs was 1.2 Å; across all 249 pairs—3.8 Å). The distance between the two Fe^3+^ ions in the aligned structures is 2.3 Å.

We produced recombinant *B. fragilis* XusB (BfXusB) and determined its crystal structure using data to 1.77 Å (Fig. 6*A*). BfXusB lacks the hook loop observed in BtXusB crystal structures (Fig. 6*B*). A previous study reported that *B. fragilis* can grow on ferrichrome as the sole iron source, but the ferrichrome transporter could not be conclusively identified (38). Ferrichrome binds iron via hydroxamate groups, rather than catecholate groups as in Ent and DGE (Fig. 6*C*). FeEnt titrations into BfXusB did not result in substantial injection heats indicating lack of binding, but titrations of ferrichrome into BfXusB showed binding (Fig. 6 *D* and *E*). Titration of ferrichrome into BtXusB and BvXusB did not show heat changes that would suggest binding (Fig. 6*E* and *SI Appendix*, Fig. S13). We then determined the structure of BfXusB from crystals soaked with ferrichrome using data to 3.32 Å resolution (Fig. 6 *F* and *G* and *SI Appendix*, Table S1). We saw additional electron density consistent with the structure of ferrichrome inside the β-propeller pocket without any changes in protein conformation compared to the apo structure (Fig. 6 *G* and *H*). The moderate resolution makes it difficult to reliably discern which residues of BfXusB interact with ferrichrome, but it is clear the binding residues are completely different to those in BtXusB and BvXusB. The side chains of L93 and W235 slot between two grooves formed by the hydroxamate arms, while the third groove remains unoccupied and exposed to solvent. The side chains of Y52, Y77, and F398 and backbones of W323 and D352-G354 interact with the cyclic peptide backbone (Fig. 6*H*), in contrast to the BtXusB–FeEnt interaction where there is a single contact with the triserine lactone backbone (Fig. 6*F*). Despite these differences, the xenosiderophore binding sites in BfXusB and BtXusB occupy the same location in the context of the DUF4374 fold (Fig. 6*I*). This points to a common evolutionary ancestry despite low sequence homology and suggests that other DUF4374-containing proteins also bind their ligands at the same site.

### Xenosiderophore Utilization Bioassay.

**Fig. 7.**
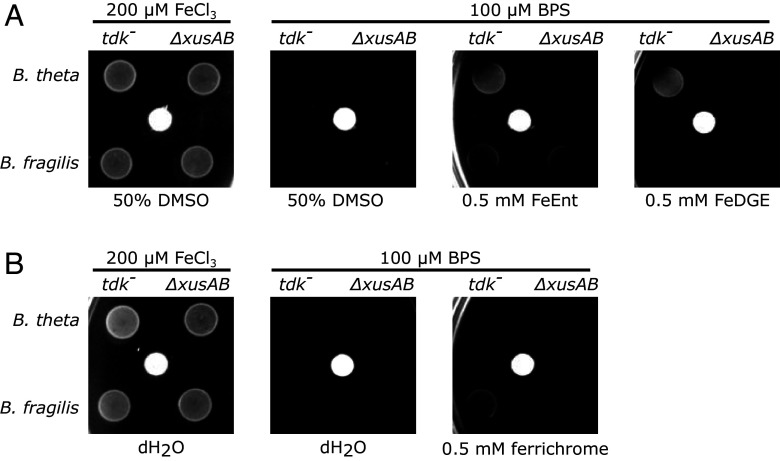
Xenosiderophore utilization bioassay. *B. theta* and *B. fragilis tdk^−^* and Δ*xusAB* strains were spotted on minimal medium agar plates under iron-replete (200 μM FeCl_3_) or iron-limiting conditions (100 μM BPS). Iron–siderophores or the solvent controls were supplied via sterile filter discs (white). The *tdk^−^* strains are thymidine kinase knockouts that are sensitive to 5-fluoro-2′-deoxyuridine that is used for counterselection during allelic exchange but otherwise have wild-type genotypes. (*A*) shows FeEnt and FeDGE (both supplied in 50% DMSO) utilization. (*B*) shows ferrichrome utilization.

The BfXusB-ferrichrome structure and binding data strongly suggested that *B. fragilis* takes up ferrichrome across its OM via a XusAB-like complex formed by BfXusB and a XusA homologue encoded in the same operon (BF9343_4229; Fig. 5*E*). We wanted to experimentally confirm this and used a modified assay by Rocha and Krykunivsky (38) to test xenosiderophore utilization in *B. theta* and *B. fragilis xusAB* deletion strains. We made *xusAB* knockouts in *B. theta* and *B. fragilis* thymidine kinase deletion (*tdk^−^*) strains using the pExchange-tdk allelic exchange system (39). As shown previously (21), *B. theta* could grow under iron-limiting conditions on both FeEnt and FeDGE as the sole iron source in a *xusAB-*dependent manner (Fig. 7*A*). We found that growth of *B. fragilis* on ferrichrome is also *xusAB*-dependent (Fig. 7*B*). This observation confirms the in vivo relevance of our in vitro findings. Surprisingly, we found that *B. fragilis* with and without the *xusAB* locus could utilize FeEnt (Fig. 7*A*). This result implies that there are more xenosiderophore utilization systems in *B. fragilis*, though BLAST searches did not reveal any paralogous loci to *xusABC* (BF9343_4229-4227).

## Discussion

There are many TonB-dependent transporters in Bacteroidota that transport unknown molecules (40). It is important to elucidate what these transporters are transporting and how, so that we can obtain a more complete understanding of microbial interactions inside the gut. Our study provides insights into xenosiderophore uptake via Bacteroidota TonB-dependent transporter–lipoprotein complexes and contributes to the ever-widening substrate range described for lipoprotein-assisted TonB-dependent transporters (2, 30, 31, 40–43).

Our cryo-EM structure shows that XusAB form a tight, closed complex even in the absence of FeEnt. It is unclear how xenosiderophores find their way to the XusB ligand binding site which faces the cavity formed by XusAB and is not easily accessible. XusB could transiently open like a lid and give access to the XusAB cavity to incoming xenosiderophores. Alternatively, the disordered β9-10 extracellular loop of XusA might act as a lateral gate for ingress of xenosiderophores. The latter hypothesis is favored by the lack of any XusAB particles in an open state in the cryo-EM data and the many interactions between the closed XusB lid and XusA (*SI Appendix*, Fig. S6). On the other hand, closure of a mobile XusB lid appears more effective in dislodging the XusB-bound siderophore via clashes with XusA loop β7-8, and we therefore favor the hypothesis of a mobile XusB lid delivering the xenosiderophore to the XusA transporter. The proposed mechanism is analogous to our previous work on BtuBG vitamin B_12_ uptake systems, where we did succeed determining cryo-EM structures of the (closed) transporter with and without B_12_. Comparison of these structures showed clashes between BtuB loops and BtuG-bound B_12_, suggesting a mechanism of tightly bound (subpicomolar affinity) B_12_ release from BtuG involving clashes with BtuB (30). Our proposed mechanism is also similar to the one established for the *Serratia marcescens* HasAR system, where the soluble hemophore HasA delivers heme to the HasR TonB-dependent transporter. In this system, HasA–heme binding to HasR results in distortion of the heme axial ligands in HasA by HasR extracellular loops and subsequent transfer of heme to HasR (24, 44).

It is noteworthy that, while open lipoprotein lids have been observed experimentally for all characterized dimeric SusCD transporters (31, 41–43), for unknown reasons this has not been the case for BtuBG (30) and XusAB systems, which are all monomeric. It is also unclear why we could not load the purified XusAB complex with FeEnt or FeDGE. The in vitro conditions may somehow preferentially stabilize the detergent-purified complex in a closed state, while in vivo the presence of negatively charged lipo-oligosaccharide in the *B. theta* OM could destabilize the XusAB interaction. It is also conceivable that OM-anchored XusB that is free and not complexed with XusA plays a role in complex opening.

We observed an excess of XusB in our native XusAB purifications (*SI Appendix*, Fig. S3). The excess XusB is likely destined for secretion in OMVs (22). It has been shown that *B. theta*-derived OMVs containing FeEnt-loaded XusB can be used as an iron source by *B. theta* and *Salmonella*, but **Bacteroides* vulgatus* (*Phocaeicola vulgatus*) could not utilize FeEnt bound to *B. theta* XusB (22). These observations show that OMVs can somehow deliver FeEnt bound to XusB to TonB-dependent transporters in the OM of recipient bacteria and that *Bacteroides* species can compete for xenosiderophores via their Xus systems. We speculate that the net flow of FeEnt is determined by the relative affinities for FeEnt of the donor XusB present in OMVs and acceptor XusAB complexes in the OM. If the former is higher than the latter, the amount of FeEnt that binds to XusAB might be insufficient to support growth of the acceptor strain.

*B. theta* XusAB can transport at least two structurally similar xenosiderophores, FeEnt and FeDGE (21, 38). BtXusB interacts extensively with the siderophore triscatecholate structure, and the binding site can also accommodate the two glucosyl groups of FeDGE. Furthermore, the triserine lactone of FeEnt and FeDGE is exposed to solvent, suggesting that the binding site could accommodate siderophores with different backbones connecting the catecholate arms, such as vibriobactin and bacillibactin, or synthetic Ent mimics (20, 45). This interaction mode raises the question of how many different xenosiderophores a single XusAB complex can import, which could have a substantial impact on our understanding of competition for iron at the host–pathogen–commensal interface.

Together, our structures, functional data, and bioinformatics analyses suggest that the presence or absence of the hook in DUF4374-containing proteins indicates preference for catecholate or hydroxamate xenosiderophores, respectively. Whether the shorter, BfXusB-like homologues can bind multiple related xenosiderophores as well remains unknown. Cyclic hydroxamate siderophores structurally related to ferrichrome, such as ferricrocin and ferrichrysin, have hydroxymethyl modifications on the peptide backbone (46–48). Because BfXusB interacts extensively with the backbone, these substitutions may either form additional contacts with BfXusB or clash with its side chains. Further functional and structural analyses are required to determine the substrate range of hydroxamate-binding XusB homologues.

While catecholate siderophores are produced by most Enterobacteriaceae, hydroxamate siderophores are mainly produced by fungi (48). Our results suggest that many Bacteroidota species, such as *B. fragilis*, can steal fungal siderophores. Interactions between bacteria and fungi in microbial communities are complex. *Candida albicans* has been shown to promote growth of *B. fragilis* in vitro (49). On the other hand, bacteria that have type VI secretion systems, including *B. fragilis,* can directly inject antifungal effectors into fungal cells (50–52). The role of iron piracy by *B. fragilis* in the context of its interactions with fungi remains unknown and requires further study.

## Materials and Methods

### Bacterial Strains and Culture Conditions.

All strains used in this study are listed in *SI Appendix*, Table S4. *E. coli* BL21(DE3) (53) and TOP10 strains were genetically manipulated using standard laboratory procedures. The kanamycin concentration used in liquid and solid lysogeny broth (LB) was 50 μg/mL. *B. theta* VPI-5482 *tdk^−^* was cultured either in brain-heart infusion (BHI, Oxoid) supplemented with 1 μg/mL hemin or defined minimal medium [50 mM potassium phosphate buffer pH 7.2, 15 mM sodium chloride, 7.5 mM ammonium sulfate, 9.4 mM sodium carbonate, 4.1 mM L-cysteine, 0.37 nM vitamin B_12_, 5.8 µM vitamin K, 180 µM calcium chloride, 100 µM magnesium chloride, 50 µM manganese(II) chloride and 42 µM cobalt(II) chloride] (54) supplemented with 1 μg/mL hemin and 0.5% fructose*. B. theta* was grown under anaerobic conditions at 37 °C in a Don Whitley A35 workstation. When required, 200 µg/mL gentamicin and 25 µg/mL erythromycin was used for selection of *B. theta.*

### Construction of Expression Plasmids.

All plasmids used in this study are listed in *SI Appendix*, Table S5. The nucleotide sequence coding for amino acid residues 35 to 464 of *B. theta* VPI-5482 XusB (*bt_2064*) and 30 to 406 of *B. fragilis* NCTC 9343 XusB (*bf9349_4228*) which excludes the signal sequence, the lipid anchor cysteine and a disordered linker, was amplified by PCR using genomic DNA as the template and primers that introduce overhangs containing NcoI and XhoI restriction sites. The PCR product was digested with NcoI and XhoI FastDigest restriction enzymes (ThermoFisher Scientific) and ligated into pET28b, resulting in a C-terminal His_6_-tag fusion. The XusB^R167A,Δhook^ binding site variant nucleotide sequence with flanking NcoI and XhoI restriction sites was synthesized and ligated into the pTwist Amp High Copy plasmid by Twist Bioscience. The synthetic DNA sequence was subcloned into pET28b using the flanking restriction sites. Similarly, the nucleotide sequence encoding amino acid residues 29-491 of *B. viscericola* DSM 18177 XusB (*BARVI_05925*) was synthesized by Twist Bioscience and subcloned into pET28b using NcoI and XhoI restriction sites. Cloning was carried out in *E. coli* TOP10 cells. Clones were screened for successful insert ligation by colony PCR using EmeraldAmp GT PCR master mix (Takara Bio) with T7 promoter and T7 terminator primers. All constructs were verified by Sanger sequencing (Eurofins).

### Construction of Chromosomal Deletions.

*B. theta* and *B. fragilis xusAB* chromosomal deletions were generated using the pExchange-tdk plasmid (39). The pExchange-tdk plasmids containing ~700 bp flanking the *xusAB* coding sequences in *B. theta* VPI-5482 (*bt2064-65*) or *B. fragilis* NCTC 9343 (*bf9343_4228-29*) were used to transform *E. coli* S-17 λ pir cells (55). The *E. coli* cells were used to introduce the pExchange-tdk plasmids into recipient *Bacteroides* strains lacking thymidine kinase (*tdk^−^*). Conjugants, which underwent a single recombination event, were selected on BHI-hemin plates containing gentamicin (200 μg/mL) and erythromycin (25 μg/mL). Single colonies were cultured in enriched BHI medium overnight, pooled, and plated on BHI-hemin agar plates containing 5-fluoro-2′-deoxyuridine (FUdR; 200 μg/mL) to select for cells that have eliminated the vector backbone from their genome in a second recombination event. After 48 h of growth, FUdR-resistant colonies were restreaked on fresh BHI-hemin-FUdR plates. Clones were screened for successful *xusAB* deletion using PCR with primers that bind outside the homologous region used to make the pExchange-tdk plasmids and confirmed by Sanger sequencing (Eurofins).

### Protein Expression and Purification in *E. coli*.

*E. coli* BL21(DE3) cells were transformed with plasmids carrying the coding sequences for soluble variants of XusB, plated on LB kanamycin agar plates and incubated at 37 °C overnight. The following day, approximately one third of transformants were scraped off the agar plate and used to start an LB kanamycin preculture, which was incubated at 37 °C with shaking for 1.5 to 3 h. 12 to 15 mL of preculture was used to inoculate 1 l flasks of prewarmed LB kanamycin medium. The cultures were incubated at 37 °C with shaking until OD_600_ reached 0.4 to 0.8. Protein expression was then induced by adding isopropyl β-d-1-thiogalactopyranoside to a final concentration of 0.1 mM, and the cultures were grown for another 16 to 20 h at 18 °C with 150 rpm shaking. Cells were harvested by centrifugation at 8,000×*g* at 4 °C for 20 min. Pellets were resuspended in cold Tris-buffered saline (TBS, 20 mM Tris-HCl, 300 mM NaCl) and stored at −20 °C until protein purification. Cell pellets were thawed and homogenized in TBS using a Dounce tissue grinder and supplemented with DNase I (Roche). Cells were lysed by passing the suspension once through a cell disruptor (Constant Systems) at 20 kpsi. The lysates were supplemented with 1 mM phenylmethylsulfonyl fluoride (PMSF) and clarified by centrifugation at 30,000×*g* for 30 min at 4 °C. The supernatants were loaded on 5 mL chelating Sepharose columns charged with Ni^2+^ ions using gravity flow. The columns were washed with 30 column volumes of TBS with 30 mM imidazole. Bound proteins were eluted with 4 column volumes of TBS with 250 mM imidazole. The eluate was concentrated with an Amicon Ultra filtration device (30 kDa cut-off membrane), loaded on a HiLoad Superdex 200 16/600 pg column and eluted in 10 mM HEPES-NaOH pH 7.5, 100 mM NaCl. Fractions were analyzed by SDS-PAGE. Fractions containing the soluble XusB variants were pooled, concentrated, flash-frozen, and stored at −80 °C.

### Synthesis of DGE.

DGE was prepared chemoenzymatically based on previously reported procedures (56, 57). A reaction solution containing 500 μM Ent and 3 mM UDP-glucose (UDP-Glc) in buffer (75 mM Tris-HCl pH 8.0, 5 mM MgCl_2_, 2.5 mM TCEP) was prepared and aliquoted into 10 × 750 μL portions. The *C-*glucosyltransferase IroB was added to each sample to a final concentration of 5 μM and the reactions were incubated at room temperature. After 1 h, each aliquot was quenched by addition of 75 μL 6% trifluoroacetic acid (TFA)/H_2_O and diluted with ∼300 μL acetonitrile (MeCN). Quenched solutions were centrifuged (13,000 rpm, 10 min, 4 °C) then purified by preparative reversed-phase HPLC (0 to 100% B in 30 min, H_2_O/MeCN + 0.1% TFA, 10 mL/min). The peak corresponding to DGE was collected and lyophilized to dryness.

### Preparation of Ferric Siderophores.

Ent and DGE were dissolved in dimethyl sulfoxide (DMSO) to make 10 mM solutions. The 10 mM siderophore solution was mixed in a 1:1 volume ratio with 10 mM aqueous FeCl_3_ solution to yield a 5 mM ferric siderophore stock solution in 50% (v/v) DMSO. The concentrations of FeEnt and FeDGE stock solutions were determined spectrophotometrically using the extinction coefficient ε_495_ = 5,600 M^−1^ cm^−1^ (in 20 mM Tris-HCl pH 7.0, 50% methanol) (58). The stock solutions were stored in small aliquots at −80 °C to minimize siderophore hydrolysis. Similarly, desferrichrome (Merck) was dissolved in sterile Milli-Q water to make a 10 mM solution, aliquoted, and stored at −20 °C. After thawing, 10 mM desferrichrome aliquots were mixed with 10 mM FeCl_3_ in a 1:1 ratio, resulting in a 5 mM aqueous ferrichrome solution. Ferrichrome concentration was confirmed spectrophotometrically using the extinction coefficient ε_425_ = 2,900 M^−1^ cm^−1^ (in 20 mM Tris-HCl pH 7.0) (59).

### Crystal Structure Determination.

The purified soluble BtXusB variant was concentrated to 36 mg/mL. Sitting drop vapor diffusion crystallization screens were set up using a Mosquito robot (SPT Labtech) either with apo BtXusB or with a 1:1.1 molar ratio of BtXusB and FeEnt. The crystallization plates were incubated at 20 °C. Apo XusB crystals appeared after less than a week in the PACT premier screen (Molecular Dimensions) containing 0.1 M MES pH 6.0, 0.2 M calcium chloride and 20% PEG 6000. Crystals were cryoprotected in mother liquor supplemented with ~20% PEG 400 and flash-cooled in liquid nitrogen. BtXusB–FeEnt cocrystals appeared in the Index screen (Hampton Research) condition containing 0.1 M citric acid pH 3.5 and 2 M ammonium sulfate after 2 wk. Crystals were cryoprotected by passing through a drop of 3.5 M ammonium sulfate and flash-cooled in liquid nitrogen. Apo BtXusB crystals grown in hanging vapor diffusion drops (0.1 M HEPES pH 6.7, 0.2 M calcium chloride, 20% PEG 6000, and 5% DMSO) were soaked with 1 mM FeDGE in mother liquor for 24 h, cryoprotected with mother liquor, 1 mM FeDGE, and 20% PEG 400, and flash-cooled in liquid nitrogen.

BvXusB was concentrated to 35 mg/mL and sitting drop crystallization trials using commercial screens were set up as above. An initial hit was observed in the Structure 1 + 2 screen (Molecular Dimensions) and further optimized using hanging drop vapor diffusion and streak seeding. The final condition for diffracting apo BvXusB crystals was 0.1 M sodium acetate pH 4.8, 0.2 M ammonium sulfate and 35% PEG 2000 monomethyl ether. The crystals were cryoprotected in mother liquor with 20% PEG 400 and flash-cooled in liquid nitrogen.

BfXusB was concentrated to 30 mg/mL and sitting drop crystallization trials using commercial screens were set up as above. Apo crystals were harvested directly from the Index screen (Hampton Research) condition containing 0.1 M HEPES pH 7.5, 0.02 M magnesium chloride hexahydrate, 22% w/v poly(acrylic acid sodium salt) 5100. Mother liquor with 20% PEG 400 was used to cryoprotect the crystals before flash-cooling in liquid nitrogen. Crystals from an identical condition in the JCSG+ screen (Molecular Dimensions) were soaked with 1 mM ferrichrome in mother liquor for 3 wk, cryoprotected in mother liquor, 1 mM ferrichrome, and 20% PEG 400 and flash-cooled in liquid nitrogen.

X-ray diffraction data were collected at the Diamond Light Source synchrotron (UK) at a temperature of −173 °C (*SI Appendix*, Table S1). Datasets were processed with XIA2-dials (60), scaled with Aimless (61), and the space group was confirmed with Pointless (62). The apo structures were solved by molecular replacement with computational models generated by AlphaFold2 (37). The siderophore-bound structures were solved by molecular replacement using the apo experimental structures. All models underwent cycles of manual building in Coot (63) and refinement in Phenix (64) until no further improvement in R factors could be achieved. The models were validated using MolProbity (65). Refinement statistics are summarized in *SI Appendix*, Table S1.

### Isothermal Titration Calorimetry.

ITC was carried out in 10 mM HEPES–NaOH pH 7.5 and 100 mM NaCl supplemented with 5% DMSO to improve FeEnt and FeDGE solubility. A 250 μM FeEnt solution was made in ITC buffer, and any precipitate was removed by centrifugation. The clarified FeEnt solution was titrated into 25 μM protein at 25 °C using a Microcal PEAQ-ITC instrument (Malvern Panalytical). After an initial delay of 60 s, a single injection of 0.4 μL was carried out, which was discarded from data analysis, followed by 18 injections of 2 μL spaced in 150 s intervals. For FeDGE titrations only, the initial 0.4 μL injection was followed by 12 injections of 3 μL spaced in 150 s intervals. The sample cell was stirred at 750 rpm during titration. Ligand to buffer control titrations were subtracted from all experiments. The experiments were repeated at least twice (*SI Appendix*, Table S2). Data were fitted to a single-binding-site model using the Microcal PEAQ-ITC Analysis software v1.40. For FeDGE titrations only, the stoichiometry (n) was fixed to one and the ligand concentration was allowed to float as the fits would not converge otherwise. We speculate that the FeDGE concentration may have been inaccurate due to hydrolysis of the siderophore during repeated freeze-thawing. Data fitting results for successful binding experiments are shown in *SI Appendix*, Table S2.

### Purification of the XusAB Complex From *B. theta*.

A C-terminal His_6_-tag was fused to XusB by introducing the tag coding sequence into the *B. theta tdk^−^* chromosome before the stop codon of *bt_2064* via allelic exchange using the pExchange plasmid (39). Presence of the tag on the chromosome was confirmed by PCR and Sanger sequencing. Conditions under which the tagged XusB is expressed were identified by Western blotting (*SI Appendix*, Fig. S3 and *SI Methods*).

Frozen *B. theta bt_2064-his* glycerol stocks were used to inoculate BHI, followed by overnight incubation at 37 °C under anaerobic conditions. After autoclaving, minimal medium was stored in an A35 Don Whitley anaerobic workstation overnight. The following day, the minimal medium was supplemented with 50 μM BPS, fructose (0.5%), and hemin (1 μg/mL). Overnight *B. theta* BHI cultures were pelleted, resuspended in minimal medium to original volume and used to inoculate 0.5 l bottles of minimal medium at a ratio of 1:250. Bacteria were cultured at 37 °C under anaerobic conditions for 18 to 20 h. Cultures were pelleted by centrifugation at 6,000×*g* for 30 min at 4 °C, resuspended in TBS, and stored at −20 °C.

Pellets were thawed, supplemented with DNase I and homogenized. Cells were lysed by passing the cell suspension once through a cell disruptor at 22 kpsi. The lysate was clarified by centrifugation at 30,000×*g*, 4 °C for 30 min. Membranes were isolated from the clarified lysate by ultracentrifugation at 140,000×*g*, 4 °C for 50 min, followed by solubilization in 1.5% lauryldimethylamine N-oxide (LDAO) in TBS for 1 h at 4 °C. Insoluble material was pelleted by centrifugation at 44,000×*g*, 4 °C for 30 min. The solubilized material was passed through ~3 mL of chelating Sepharose resin charged with Ni^2+^ ions using gravity flow. The column was washed with 25 column volumes of TBS with 30 mM imidazole and 0.1% dodecyl-β-D-maltopyranoside (DDM, Anatrace), and bound protein was eluted with TBS supplemented with 200 mM imidazole and 0.03% DDM. The eluate was concentrated using an Amicon Ultra filtration device (100 kDa cut-off membrane), loaded on a Superdex 200 10/300 Increase column and eluted in 10 mM HEPES-NaOH pH 7.5, 100 mM NaCl, 0.03% DDM. Fractions corresponding to the XusAB peak were pooled, concentrated, flash-frozen in liquid nitrogen, and stored at −80 °C.

### Cryo-EM Structure Determination.

Pure XusAB complex at 7 mg/mL was incubated with a fourfold molar excess of FeEnt for 45 min at room temperature. 3.5 μL of the complex was then applied to glow-discharged Quantifoil R1.2/1.3 copper 200 mesh holey carbon grids. The grids were immediately blotted for 1 to 5 s and plunge-frozen in liquid ethane using a Vitrobot Mark IV (ThermoFisher Scientific) device operating at 4 °C and 100% humidity. The grids were initially screened on a 200 kV FEI Glacios microscope at the University of York (UK). Data were collected at the Astbury Centre (Leeds, UK) on a FEI Titan Krios microscope operating at 300 kV using a Falcon 4i direct electron detector (ThermoFisher Scientific) operating in counting mode (*SI Appendix*, Table S3). A total of 6,645 movies were recorded in electron event representation (EER) format at 165,000× magnification, corresponding to a pixel size of 0.74 Å.

The cryo-EM workflow is shown in *SI Appendix*, Fig. S4. Data processing was done in cryoSPARC v4.4.1 and v4.7.1 (66). Movies were motion-corrected using patch motion correction. CTF parameters were fitted using patch CTF correction. Initially, ~2,000 particles were picked manually and used to make 2D classes for template picking. 1,110,843 picked particles were extracted in 480-pixel boxes (0.74 Å/pixel), Fourier cropped to a box size of 240 pixels (1.48 Å/pixel) and subjected to two rounds of 2D classification. Ab initio models were generated from classes exhibiting protein density and decoy models were generated from classes containing predominately noise. Particles selected after 2D classification were subjected to heterogeneous refinement against four ab initio models, two of which were decoys. A single class from heterogeneous refinement refined to high resolution in nonuniform refinement (67). Most particles clustered into a single class in 3D classification (8 classes in total). Particles from this class were re-extracted in 480-pixel boxes (0.74 Å/pixel) and refined using nonuniform refinement (per-particle defocus, tilt and trefoil refinement enabled), followed by local refinement. Per-particle motion correction was performed using reference-based motion correction, followed by local refinement and re-estimation of per-particle defocus, tilt and trefoil parameters. The particle stack was subjected to a final round of 2D classification with the noise model (sigma) annealing turned off to remove any remaining poor-quality particles. Another round of reference-based motion correction was performed, followed by a final round of local refinement that gave a map with a global resolution of 2.7 Å based on the FSC=0.143 criterion. The final stack had 76,979 particles. An orientation diagnostics job was used to ascertain that the volume does not suffer from orientation bias defects due to nonuniform particle viewing angle distribution (*SI Appendix*, Fig. S5).

The XusAB protein sequences were supplied to ModelAngelo (68) for automated model building. The resulting model was iteratively adjusted in Coot (63) and ISOLDE (69) and refined using Phenix real space refinement (64). The model was validated using MolProbity (65). Refinement statistics are shown in *SI Appendix*, Table S3.

### Structure Analysis and Visualization.

Atomic models, electron density maps, and cryo-EM maps were analyzed in Coot (63) and UCSF ChimeraX (70). All figures depicting structural data were generated using UCSF ChimeraX. The ISOLDE (69) plugin was used to visualize electron density maps.

### Xenosiderophore Utilization Bioassay.

*B. theta tdk^−^*, *B. theta ΔxusAB*, *B. fragilis tdk^−^*, and *B. fragilis ΔxusAB* strains were grown overnight in enriched BHI (EBHI; 37 g/L Oxoid BHI powder, 5 g/L yeast extract) supplemented with 1 μg/mL hemin. 0.3 mL of the overnight cultures were used to inoculate 5 mL of fresh EBHI-hemin medium. The strains were cultured anaerobically at 37 °C for 3.5 h. Cells were pelleted, resuspended in 5 mL fresh minimal medium with hemin and 0.5% fructose, and diluted to OD ~ 0.05. 10 μL aliquots were spotted on minimal medium agar plates supplemented with 0.5% fructose, 10 μg/mL protoporphyrin IX and either 200 μM FeCl_3_ or 100 μM BPS. The spots were placed 1.5 cm from the center of sterile filter discs embedded in agar. Four 5 μL drops of 0.5 mM FeEnt in 50% DMSO, 0.5 mM FeDGE in 50% DMSO, 0.5 mM ferrichrome in sterile water, 50% DMSO only, or sterile water only were added to the filter discs. The plates were incubated at 37 °C anaerobically for 18 h before imaging.

## Supplementary Material

Appendix 01 (PDF)

Movie S1. Morph of the apo BtXusB crystal structure to the FeEnt-bound BtXusB crystal structure. The protein model is depicted as a cartoon and as a surface. The siderophore-binding loops are in blue. The position of FeEnt, shown as an orange stick model, is fixed for reference.

Movie S2. Fit of FeEnt and the BtXusB residues and interacting water molecules to the 2mF_o_-DF_c_ electron density map contoured at 1.5σ. FeEnt is in orange, BtXusB residues are in grey, and water molecules are shown as red spheres.

## Data Availability

Electron microscopy volumes of the XusAB complex have been deposited in the Electron Microscopy Data Bank with the accession code EMD-51210 (71), and the atomic coordinates have been deposited in the Protein Data Bank under the accession code 9GBC (72). Atomic coordinates and the associated crystallographic structure factors have been deposited in the Protein Data Bank under the following accession codes: 9GCY (apo BtXusB) (73), 9GCZ (BtXusB–FeEnt) (74), 9HQ1 (BtXusB–FeDGE) (75), 9GAR (apo BvXusB) (76), 9HQE (apo BfXusB) (77), and 9HQK (BfXusB-ferrichrome) (78).
