# Supplementary material for: Structural basis of iron piracy by human gut Bacteroides
Source: Proc Natl Acad Sci U S A. 2026 May 1;123(18):e2528036123. doi: 10.1073/pnas.2528036123 (PMC13142918; doi:10.1073/pnas.2528036123)
Supplement: Supplementary file 1 — Appendix 01 (PDF) [file pnas.2528036123.sapp.pdf]

PNAS

Supporting Information for
Structural basis of iron piracy by human gut *Bacteroides*

Augustinas Silale[1]*, Yung Li Soo[1], Hannah Mark[1], Rachel N. Motz[2], Arnaud Baslé[1], Elizabeth M. Nolan[2], Bert van den Berg[1]*

[1]Biosciences Institute, Newcastle University, Framlington Place, Newcastle upon Tyne, NE2 4HH, United Kingdom.
[2]Department of Chemistry, Massachusetts Institute of Technology, Cambridge, Massachusetts 02139, United States.

*Correspondence to: Augustinas Silale, Bert van den Berg

Email: augustinas.silale@newcastle.ac.uk, bert.van-den-berg@newcastle.ac.uk

This PDF file includes:

Figures S1 to S12
Tables S1 to S5
Legends for Movies S1 to S2
SI Methods
SI References

Other supporting materials for this manuscript include the following:

Movies S1 to S2

A

FeEnt			BtXusB			Interface area (Å^2)	$\Delta^i G$, kcal/mol	N_{HB}	N_{SB}	CSS
$^iN_{at}$	$^iN_{res}$	Surface (Å^2)	$^iN_{at}$	$^iN_{res}$	Surface (Å^2)					
42	N/A	718	59	16	17,327	418.8	3.1	10	0	0.005

B

FeEnt	Dist. (Å)	BtXusB
EB4 802 (N2)	3.13	GLN 413 (OE1)
EB4 802 (O2)	3.57	GLN 413 (OE1)
EB4 802 (O4)	2.44	SER 121 (OG)
EB4 802 (O5)	2.57	SER 168 (OG)
EB4 802 (O6)	2.59	TYR 411 (OH)
EB4 802 (O15)	3.40	ASN 120 (ND2)
EB4 802 (O4)	3.33	SER 121 (N)
EB4 802 (O4)	3.23	SER 168 (N)
EB4 802 (O5)	3.02	SER 168 (N)
EB4 802 (O7)	2.92	ARG 255 (NE)

Fig. S1. PISA(1) **analysis of BtXusB-FeEnt interactions.** (**A**) Complex surface area calculations. (**B**) Hydrogen bonds formed between FeEnt and BtXusB.

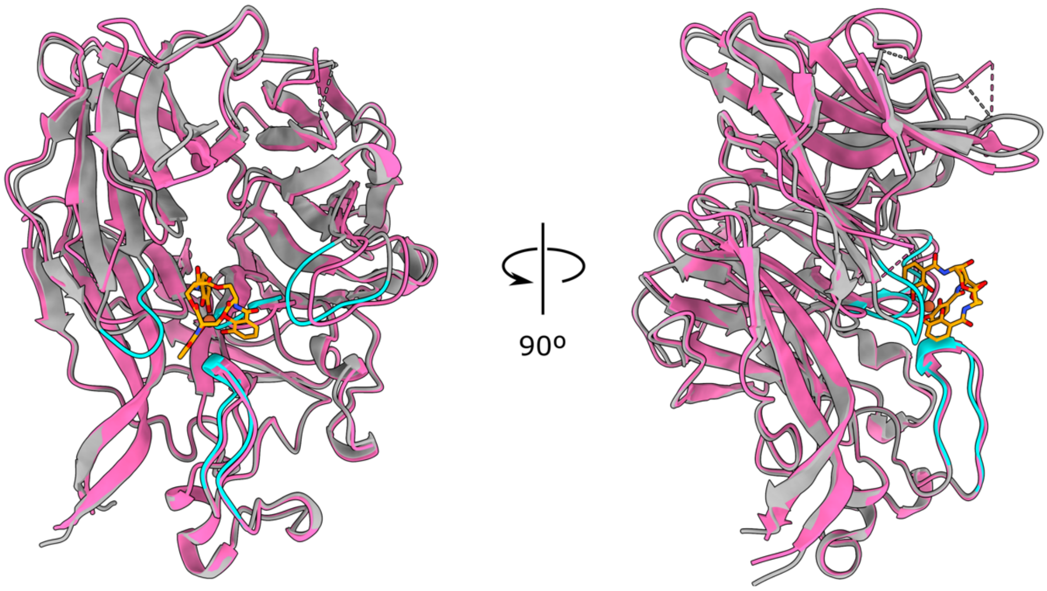

Fig. S2. Comparison of apo and FeEnt-bound BtXusB structures. Structural alignment of apo (hot pink) and FeEnt-bound (grey) BtXusB crystal structures. Cα-Cα RMSD = 1.07 Å. FeEnt is in orange; BtXusB FeEnt-binding loops are highlighted in cyan in the co-crystal structure.

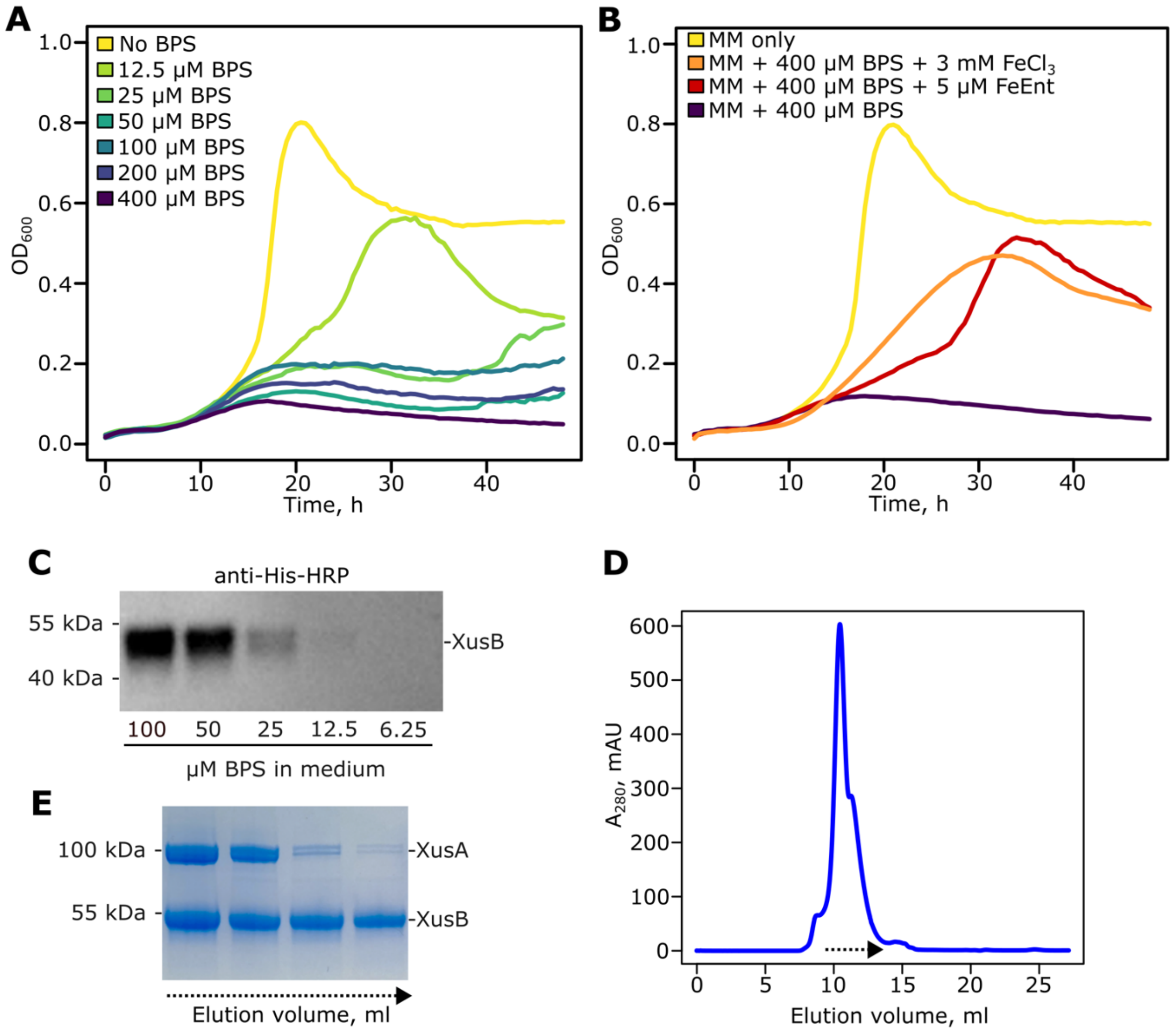

Fig. S3. Expression and purification of the native XusAB complex. (**A**) *B. theta tdk⁻* strain grown anaerobically in minimal medium with 0.5% fructose and 1 μg/ml hemin (SI Methods). The iron chelator bathophenanthroline disulfonate (BPS) was added at indicated concentrations. (**B**) *B. theta tdk⁻* strain grown anaerobically in the presence of BPS and indicated iron sources. All growth curves shown are averages from 3 wells of a single 96-well growth experiment. The experiment was repeated three times under identical conditions with similar results. (**C**) Western blot of whole cell lysates from *B. theta bt2064-his* cells grown overnight in minimal medium and the indicated concentrations of BPS (SI Methods). (**D**) Size exclusion chromatography trace of XusAB complex after immobilized metal affinity chromatography on a Superdex 200 10/300 Increase column. (**E**) SDS-PAGE analysis of 0.5 ml elution fractions from (**D**) indicated by the dashed arrow (approximately 10-12 ml elution volume). The indicated bands were identified as XusA and XusB by peptide mass fingerprinting. Both bands around 100 kDa correspond to XusA. These data suggest there is an excess of XusB relative to XusA in the OM, which might be packaged into outer membrane vesicles (2).

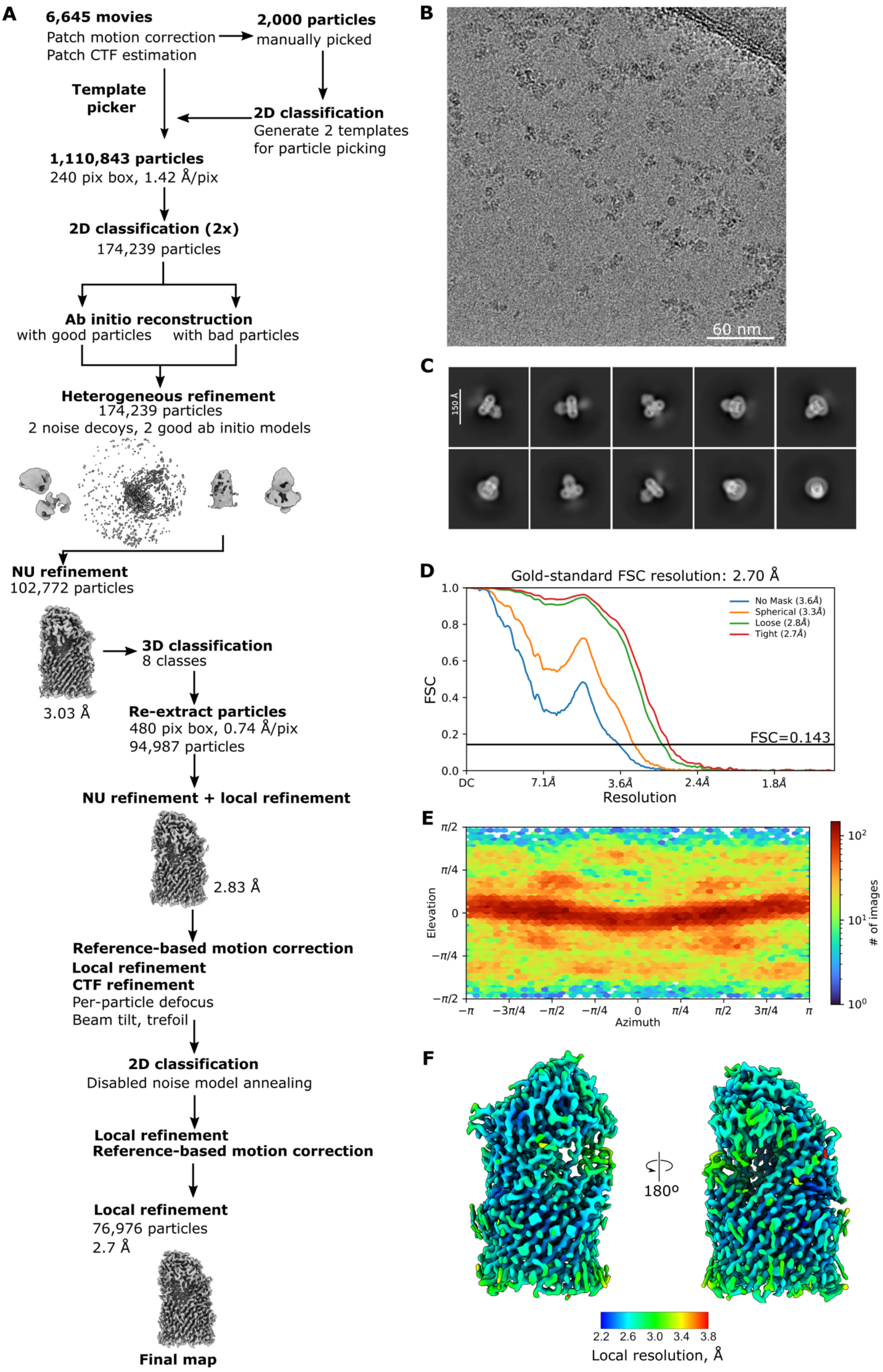
A
6,645 movies
Patch motion correction
Patch CTF estimation
2,000 particles
manually picked
Template picker
2D classification
Generate 2 templates for particle picking
1,110,843 particles
240 pix box, 1.42 Å/pix
2D classification (2x)
174,239 particles
Ab initio reconstruction
with good particles
with bad particles
Heterogeneous refinement
174,239 particles
2 noise decoys, 2 good ab initio models
NU refinement
102,772 particles
3.03 Å
3D classification
8 classes
Re-extract particles
480 pix box, 0.74 Å/pix
94,987 particles
NU refinement + local refinement
2.83 Å
Reference-based motion correction
Local refinement
CTF refinement
Per-particle defocus
Beam tilt, trefoil
2D classification
Disabled noise model annealing
Local refinement
Reference-based motion correction
Local refinement
76,976 particles
2.7 Å
Final map
B
60 nm
C
150 Å
D
Gold-standard FSC resolution: 2.70 Å
No Mask (3.6Å)
Spherical (3.3Å)
Loose (2.8Å)
Tight (2.7Å)
FSC
FSC=0.143
DC
7.1Å
3.6Å
2.4Å
1.8Å
Resolution
E
Elevation
Azimuth
# of images
F
180°
Local resolution, Å
2.2 2.6 3.0 3.4 3.8

Fig. S4. XusAB cryo-EM data processing. (**A**) Data processing workflow carried out in cryoSPARC v4.4.1(3). (**B**) Representative motion-corrected micrograph (n=6,645). (**C**) Representative 2D class averages. (**D**) Gold-standard Fourier shell correlation curve and (**E**) viewing direction distribution plot for the final map. (**F**) The final cryo-EM map coloured by estimated local resolution.

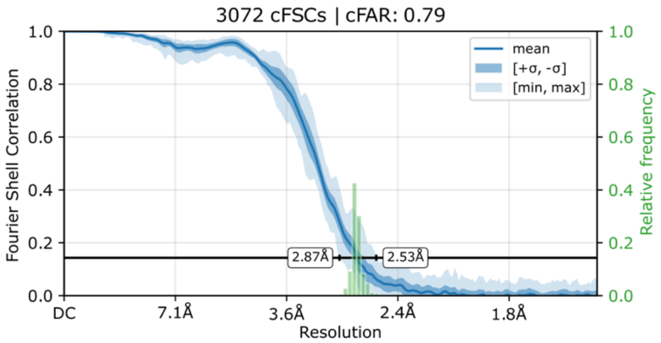

Fig. S5. XusAB cryo-EM map orientation diagnostics. Output from an orientation diagnostics job from cryoSPARC v.4.7.1. A cFAR value of >0.5 indicates that there is no orientation bias. cFSC, conical Fourier shell correlation; cFAR, conical FSC area ratio.

A

XusB			XusA			Interface area (Å^2)	Δ^iG, kcal/mol	N_{HB}	N_{SB}	CSS
$^iN_{at}$	$^iN_{res}$	Surface (Å^2)	$^iN_{at}$	$^iN_{res}$	Surface (Å^2)					
327	98	18,536	292	81	32,277	2,794.6	-12.4	52	6	1.000

B

XusB	Dist. (Å)	XusA
THR 438 (OG1)	3.10	GLU 289 (OE2)
MET 54 (N)	3.21	SER 297 (O)
GLU 436 (N)	3.02	ILE 299 (O)
LYS 432 (NZ)	3.83	THR 301 (OG1)
VAL 59 (N)	3.14	LYS 416 (O)
ASN 412 (ND2)	3.70	GLU 421 (O)
GLN 413 (NE2)	3.85	SER 423 (OG)
ARG 255 (NH2)	3.47	ASP 568 (O)
ARG 167 (NH2)	3.04	GLU 621 (OE2)
GLY 258 (N)	3.57	THR 623 (O)
ALA 259 (N)	3.60	THR 623 (O)
GLY 258 (N)	3.31	SER 624 (O)
THR 260 (OG1)	2.88	ASN 625 (OD1)
THR 249 (OG1)	3.23	ASN 625 (OD1)
THR 310 (N)	2.95	THR 674 (O)
THR 310 (N)	3.81	THR 674 (OG1)
ASN 387 (ND2)	3.20	GLU 728 (O)
ASN 387 (N)	3.16	ALA 729 (O)
ASN 386 (N)	3.43	ALA 729 (O)
ASN 387 (ND2)	2.82	GLY 731 (O)
GLU 391 (N)	3.20	THR 735 (OG1)
MET 54 (O)	3.11	SER 297 (N)
GLU 436 (O)	3.13	ILE 299 (N)
SER 434 (O)	3.33	THR 301 (N)
GLU 436 (OE1)	2.84	THR 301 (OG1)
GLU 436 (OE1)	3.15	LYS 354 (NZ)

XusB	Dist. (Å)	XusA
ASN 56 (O)	3.05	LYS 416 (NZ)
ARG 57 (O)	2.80	ARG 418 (NH1)
ARG 57 (O)	2.90	ARG 418 (NH2)
ILE 44 (O)	3.00	ARG 418 (NH2)
ASP 459 (O)	3.86	LYS 419 (N)
ASP 194 (OD1)	3.15	SER 516 (OG)
ASP 194 (OD1)	3.22	THR 517 (OG1)
ILE 192 (O)	3.11	THR 517 (OG1)
ASP 194 (OD1)	3.54	SER 518 (N)
ASP 194 (OD1)	2.84	SER 518 (OG)
ASP 194 (OD2)	3.08	THR 520 (OG1)
ASP 194 (OD2)	3.50	SER 521 (N)
SER 289 (O)	3.00	THR 623 (N)
ALA 259 (O)	3.69	THR 623 (OG1)
SER 289 (O)	3.79	THR 623 (OG1)
SER 289 (O)	3.47	SER 624 (N)
PRO 308 (O)	2.55	THR 674 (OG1)
PRO 308 (O)	3.30	GLN 676 (NE2)
ASN 316 (O)	2.81	GLN 676 (NE2)
THR 310 (OG1)	3.17	GLN 676 (NE2)
PRO 384 (O)	3.29	GLY 731 (N)
GLY 389 (O)	2.87	ALA 732 (N)
GLY 389 (O)	3.66	THR 735 (OG1)
GLU 391 (OE2)	2.99	LYS 736 (NZ)
GLU 391 (OE1)	3.57	ASP 737 (N)

C

XusB	Dist. (Å)	XusA
ARG 167 (NH2)	3.04	GLU 621 (OE2)
GLU 436 (OE2)	3.39	LYS 354 (NZ)
GLU 436 (OE1)	3.15	LYS 354 (NZ)
GLU 416 (OE2)	3.12	LYS 354 (NZ)
GLU 391 (OE1)	3.95	LYS 736 (NZ)
GLU 391 (OE2)	2.99	LYS 736 (NZ)

Fig. S6. PISA(1) **analysis of XusA-XusB interactions.** (**A**) Complex surface area calculations. (**B**) Hydrogen bonds formed between XusA and XusB. (**C**) Salt bridges formed between XusA and XusB.

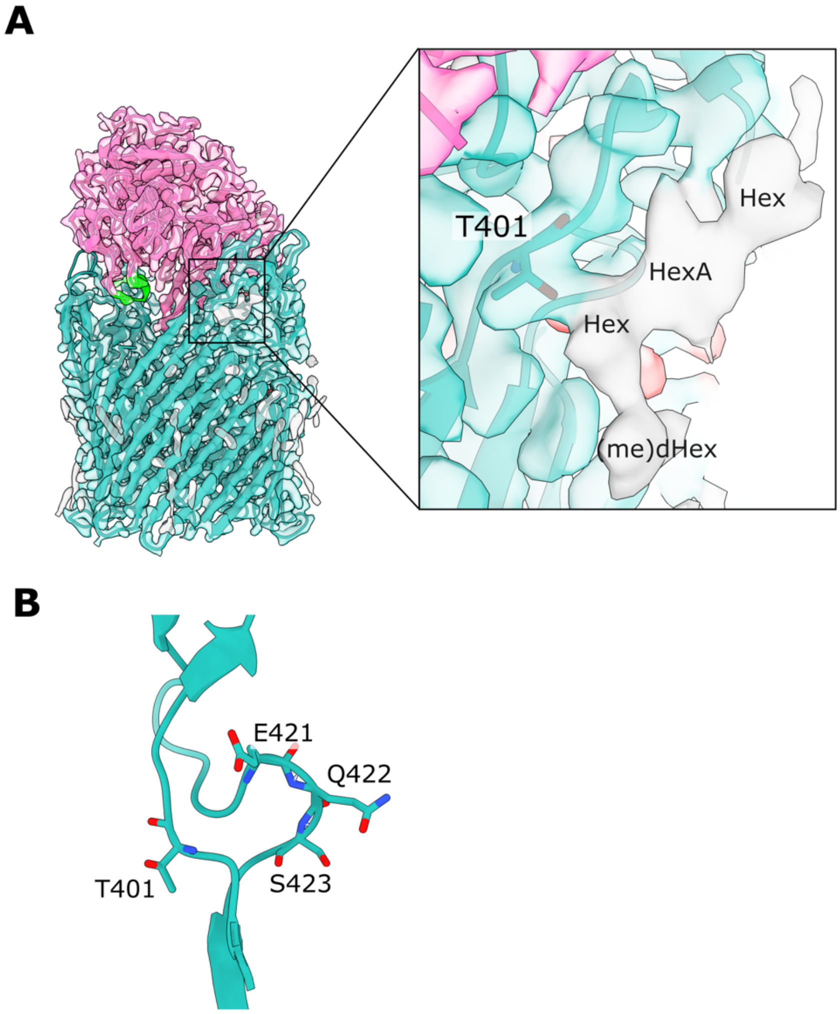

Fig. S7. XusA O-glycosylation. (**A**) The glycan density extending from the sidechain hydroxy oxygen of T401 is shown in grey. Sugar units that likely constitute the glycan chain are indicated: Hex, hexose; HexA, hexuronic acid; (me)dHex, (methyl)deoxyhexose. Sugars were assigned based on the structure of the *B. fragilis* O-glycan(4). (**B**) Residues 421-423, which are in the C-terminal part of the β7-8 extracellular loop of XusA and are implicated in FeEnt release from XusB, are somewhat close to T401. The sidechain of T401 faces away from residues 421-423 towards the solvent.

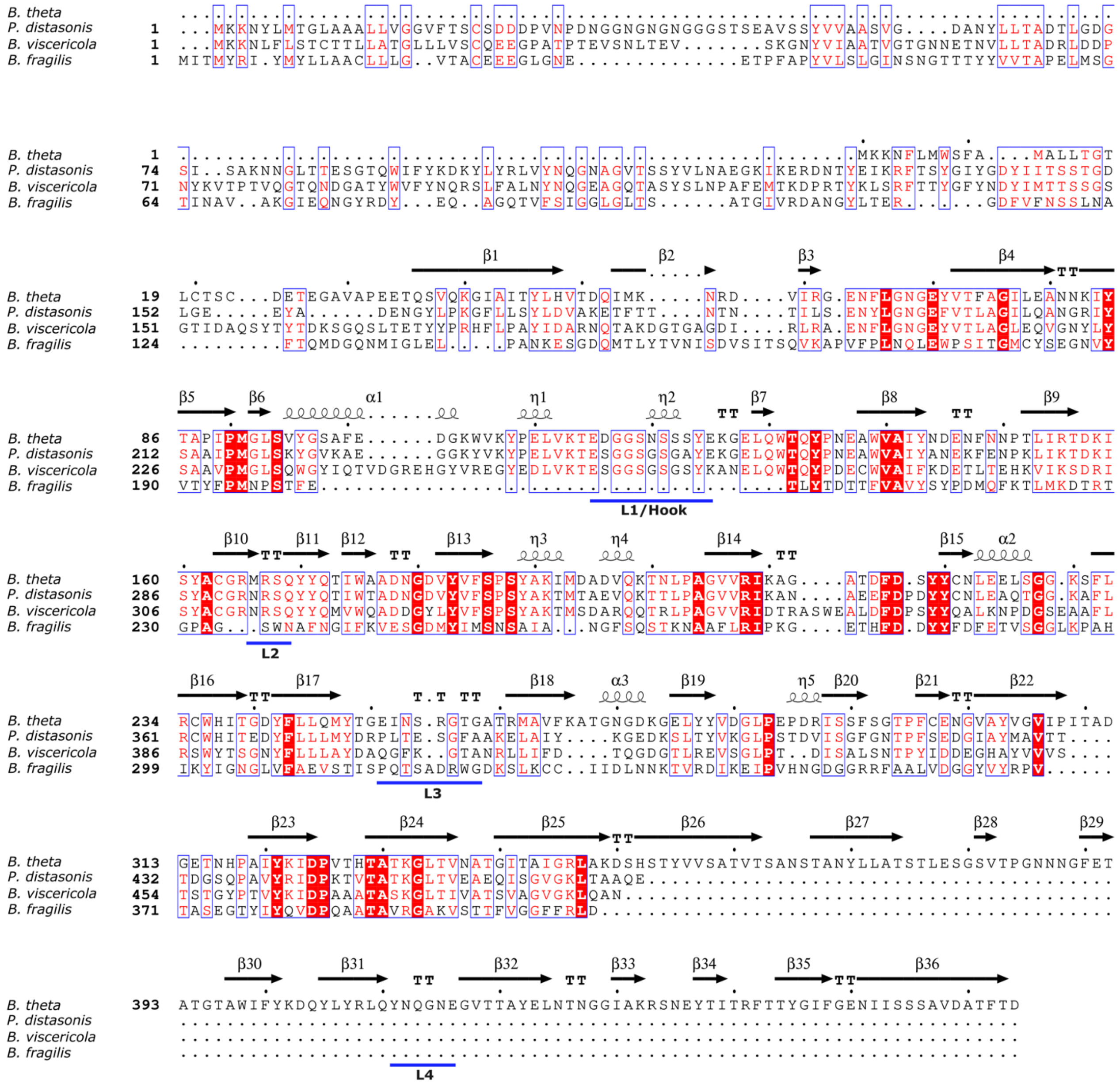

Fig. S8. XusB homologue sequence alignment. Alignment of BtXusB, *P. distasonis* DSM 20701 BDI_3402, *B. viscericola* DSM 18177 BARVI_05925, and *B. fragilis* NCTC 9343 BF9343_4228 amino acid sequences. The secondary structure features of BtXusB in the BtXusB-FeEnt structure are annotated above the alignment. Visualised in ESPript v3.0 (5) (https://espript.ibcp.fr).

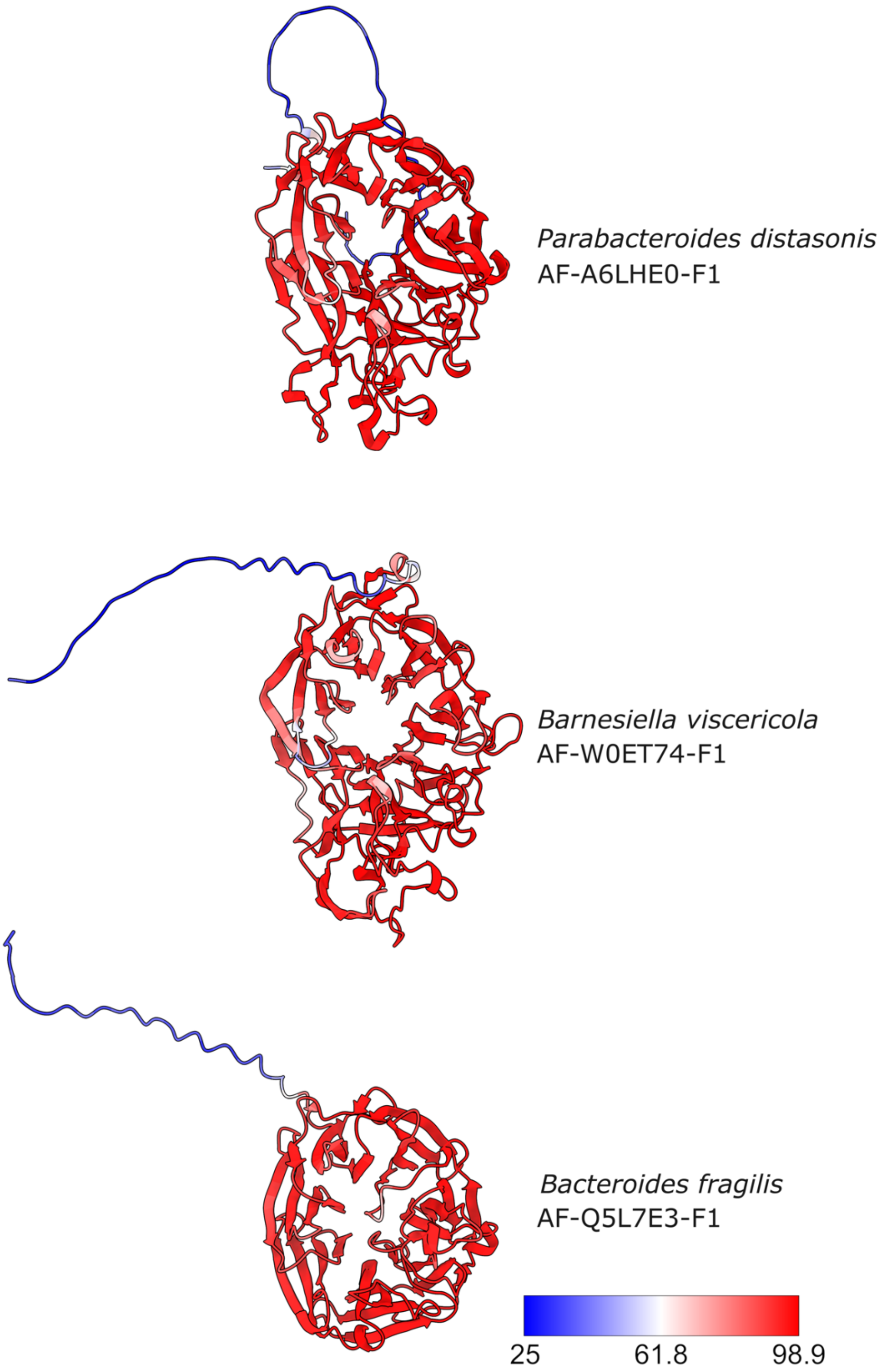

Fig. S9. XusB homologue AlphaFold2(6) **model prediction confidence.** The indicated entries were downloaded from AlphaFold DB. The views were generated from superposition with the BtXusB-FeEnt crystal structure. Each model is coloured according to pLDDT value (colour key). The blue (low confidence) N-terminal regions correspond to the signal peptides.

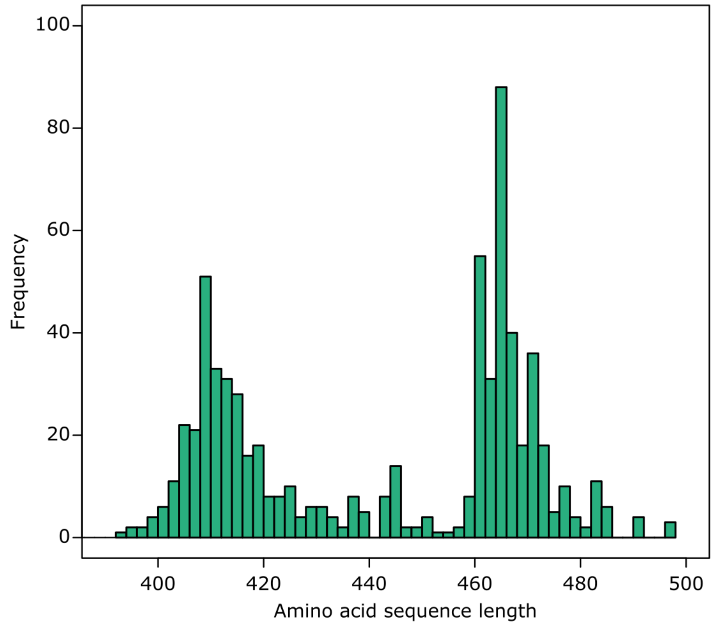

Fig. S10. XusB BLAST hit amino acid sequence length distribution. The BtXusB amino acid sequence was submitted to the EFI Enzyme Similarity Tool server(7). BLAST results were filtered to remove hits with an E-value higher than 10^{-5} and sequences shorter than 380 and longer than 500 amino acids, resulting in 681 hits. The amino acid sequence lengths of these hits are plotted in the histogram, clearly showing a bimodal distribution with peaks around 410 and 465 amino acids. The shorter group includes BfXusB, while BtXusB, BvXusB and *P. distasonis* XusB belong to the longer group.

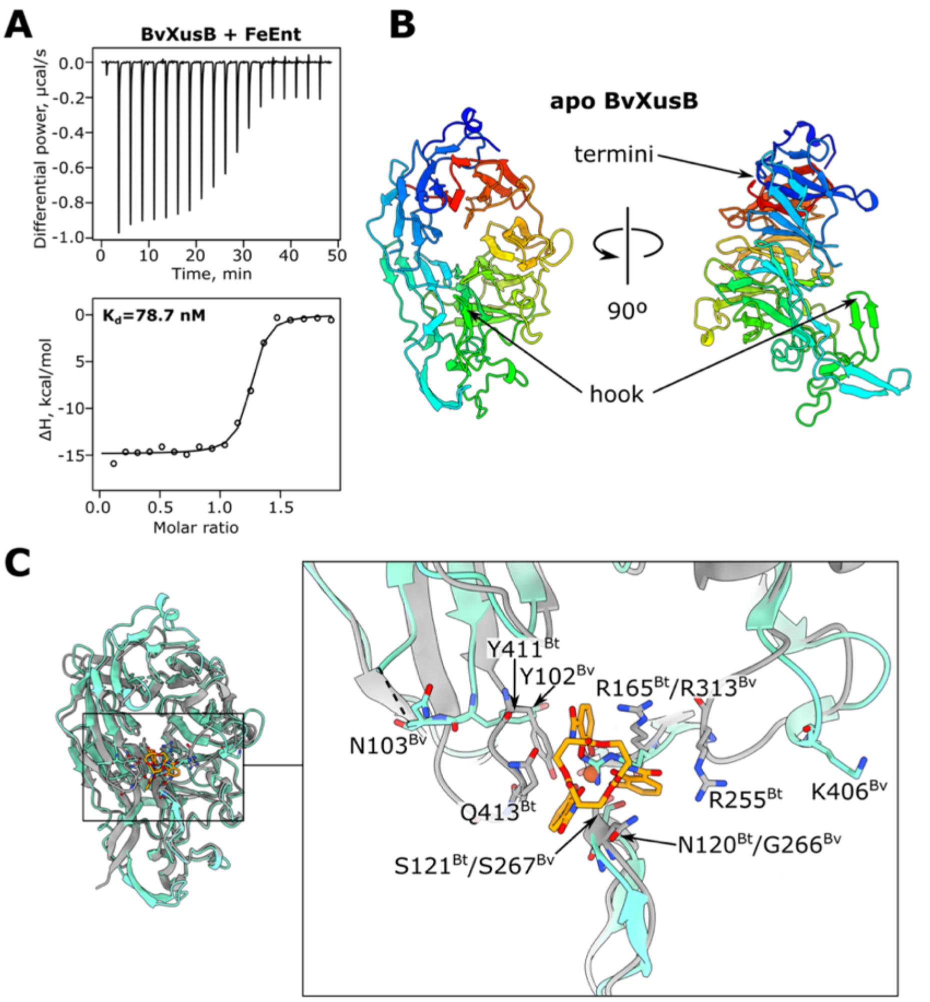

Fig. S11. ***B. viscericola*** **XusB binds FeEnt.** (**A**) Representative ITC experiment where 250 µM FeEnt was titrated into 25 µM BvXusB (n=2). Integrated heats were fitted to a single binding site model, giving the apparent K_d value. (**B**) Crystal structure of apo BvXusB to 3.2 Å. (**C**) Comparison of apo BvXusB (cyan) and BtXusB-FeEnt (grey and orange) crystal structures. The models were superposed using the Matchmaker tool in ChimeraX (Cα-Cα RMSD between 252 pruned atom pairs was 1.0 Å; across all 301 pairs—3.6 Å). BtXusB residues involved in FeEnt binding and the equivalent residues in BvXusB are shown as stick models. The dashed line indicates a disordered loop in the BvXusB crystal structure.

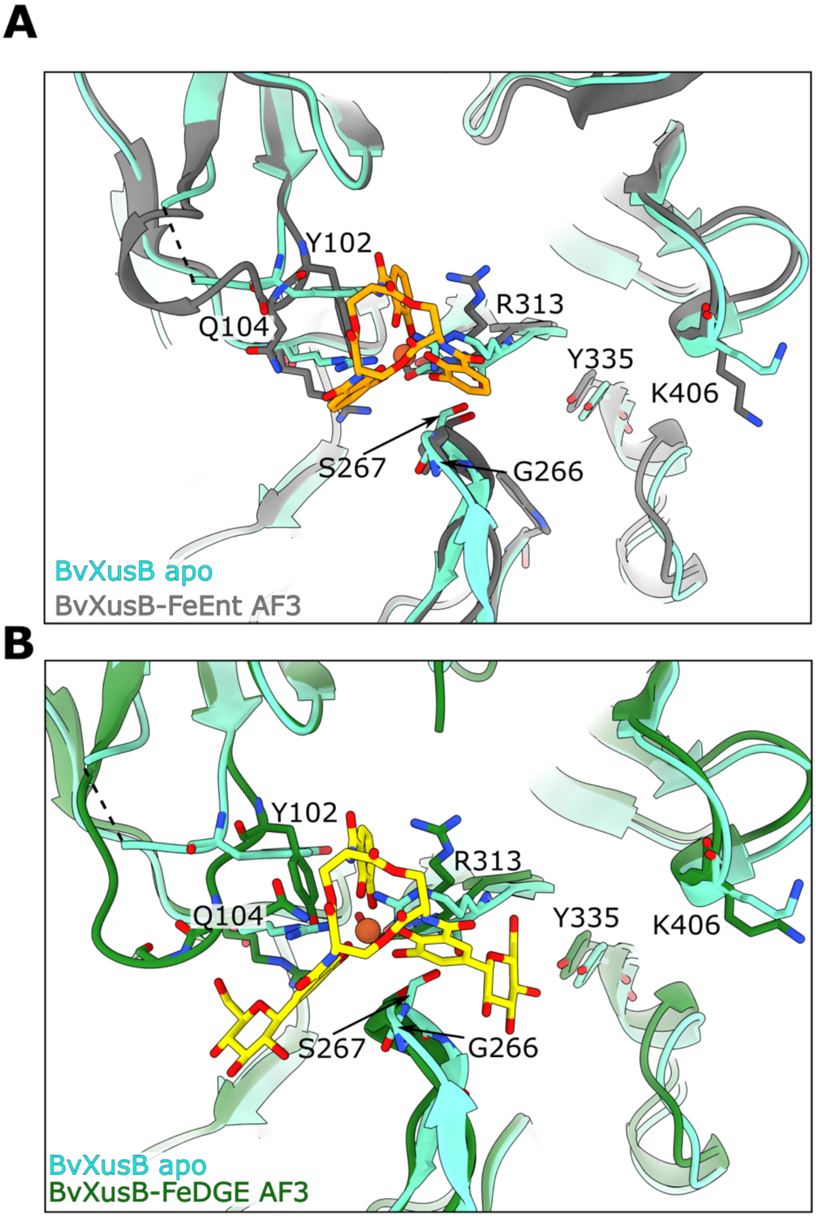

Fig. S12. Comparison of BvXusB apo structure and ligand-bound structure predictions. BvXusB apo crystal structure (cyan) superposed with AF3-predicted BvXusB-FeEnt (**A**) and BvXusB-FeDGE (**B**) models.

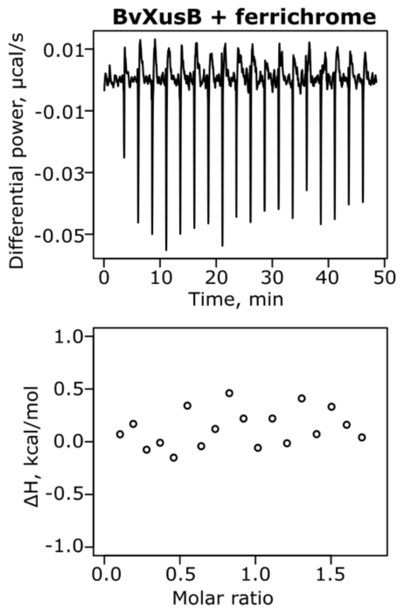

Fig. S13. BvXusB does not bind ferrichrome. Representative ITC experiment where 250 µM ferrichrome was titrated into 25 µM BvXusB (n=2). No substantial injection heats were observed, consistent with lack of binding.

Table S1. X-ray data collection and refinement parameters. Values for highest resolution shell in parentheses.

	**Apo BtXusB**	**BtXusB-FeEnt**	**BtXusB-FeDGE**	**Apo BvXusB**	**Apo BfXusB**	**BfXusB-ferrichrome**
**Data collection**						
DLS beamline	I03	I24	I03	I03	I03	I03
Wavelength	0.9537	0.6199	0.9686	0.9686	0.9686	0.9686
Space Group	$P2_1$	$P2_1$	$P2_1$	$I4_122$	$P2_12_12_1$	$P2_1$
Unit cell parameters						
a, b, c (Å)	45.7, 108.63, 93.89	59.99, 48.18, 135.47	45.44, 108.71, 93.83	184.87, 184.87, 82.31	50.99, 86.93, 154.39	57.20, 155.97, 89.62
α, β, γ (°)	90, 96.57, 90	90, 96.79, 90	90, 96.66, 90	90, 90, 90	90, 90, 90	90, 95.56, 90
Molecules in AU	2	2	2	1	2	4
Resolution range (Å)	54.32-1.56 (1.59-1.56)	39.17-1.50 (1.53-1.50)	54.35-1.80 (1.83-1.80)	58.46-3.2 (3.43-3.2)	48.42-1.77 (1.81-1.77)	58.71-3.32 (3.59-3.32)
I/σI	11.0 (1.2)	6.7 (1.4)	9.4 (1.2)	6.8 (1.8)	15.2 (2.0)	5.3 (2.4)
Completeness (%)	100 (99.5)	99.6 (94.4)	99.9 (99.4)	99.9 (99.3)	100 (99.9)	100 (100)
Multiplicity	6.8 (5.3)	6.7 (4.9)	6.8 (6.9)	27.3 (28.3)	13.4 (13.9)	7.1 (7.4)
R_{pim}	0.039 (0.652)	0.096 (0.955)	0.045 (0.468)	0.104 (0.444)	0.029 (0.355)	0.116 (0.318)
R_{merge}	0.091 (1.283)	0.162 (1.049)	0.110 (1.151)	0.538 (2.335)	0.102 (1.289)	0.290 (0.812)
$CC_{1/2}$	0.998 (0.621)	0.774 (0.545)	0.998 (0.609)	0.998 (0.751)	0.999 (0.799)	0.981 (0.862)
No. of unique reflections	129,093 (6,316)	122,900 (5,684)	83,651 (4,580)	12,010 (2,123)	67,890 (3,826)	23,146 (4,780)
**Phasing**						
Molecular replacement model	AlphaFold2 model (AF-Q8A622-F1)	apo XusB structure (PDB 9GCY)	apo XusB structure (PDB 9GCY)	AlphaFold2 model (AF-W0ET74-F1)	AlphaFold2 model (AF-Q5L7E3-F1)	Apo BfrXusB structure (PDB 9HQE)
**Refinement**						
Resolution (Å)	46.94-1.56	33.94-1.50	54.36-1.80	49.33-3.20	48.42-1.77	58.71-3.32
Rwork/Rfree	0.1757/0.2068	0.2038/0.2439	0.1916/0.2282	0.2338/0.2768	0.1987/0.2368	0.2484/0.2958
Reflections	128,930	121,657	83,497	11,963	67,737	22,931
Non-hydrogen atoms	7,736	7,583	7,471	3,594	6,081	11,812
Protein only	6,522	6,585	6,605	3,585	5,708	11,616
Mean B-factor (Å^2)	25.41	17.44	26.00	64.54	32.43	51.06
Rmsd						
Bond lengths (Å)	0.010	0.006	0.007	0.001	0.006	0.003
Bond angles (°)	0.99	0.80	0.81	0.39	0.79	0.63
Clashscore	1.94	2.14	2.00	4.29	3.01	8.45
Rotamer outliers (%)	0.14	0.71	0.85	0	0.16	0
Ramachandran plot						
Favoured (%)	96.90	97.26	96.79	95.15	94.38	94.00
Outliers (%)	0	0	0.12	0	0.14	0.27
PDB ID	9GCY	9GCZ	9HQ1	9GAR	9HQE	9HQK

Table S2. Isothermal titration calorimetry data fitting results.

Titration	Replicate	$[Protein]_{cell}$ (M)	$[Ligand]_{syringe}$ (M)	N	K_d (M)	ΔH (kcal/mol)	ΔG (kcal/mol)	-TΔS (kcal/mol)
BtXusB + FeEnt	1	2.50E-05	2.50E-04	1.3 ± 4.90E-03	9.82E-08 ± 1.19E-8	-24.5	-9.57	14.9
BtXusB + FeEnt	2	2.50E-05	2.50E-04	1.46 ± 3.50E-03	8.42E-08 ± 7.02E-08	-22.8	-9.67	13.1
BtXusB + FeEnt	3	2.50E-05	2.50E-04	1.47 ± 4.90E-03	4.94E-08 ± 7.45E-09	-21	-9.97	11
BtXusB + FeEnt	4	2.50E-05	2.50E-04	1.22 ± 4.10E-03	5.52E-08 ± 8.00E-09	-22.6	-9.9	12.7
BtXusB + FeDGE	1	1.73E-05	1.54E-04	1 (fixed)	1.69E-07 ± 2.10E-08	-20.1	-9.24	10.8
BtXusB + FeDGE	2	1.73E-05	1.57E-04	1 (fixed)	1.99E-07 ± 1.70E-8	-19.5	-9.14	10.4
BvXusB + FeEnt	1	2.50E-05	2.50E-04	1.35 ± 8.70E-03	1.01E-07 ± 2.11E-08	-15.5	-9.55	5.98
BvXusB + FeEnt	2	2.50E-05	2.50E-04	1.21 ± 8.80E-03	7.87E-08 ± 2.05E-08	-14.8	-9.69	5.15
BfXusB + ferrichrome	1	2.50E-05	2.50E-04	1.18 ± 2.00E-02	2.68E-07 ± 8.56E-08	-11.5	-8.97	2.52
BfXusB + ferrichrome	2	2.54E-05	3.48E-04	1.27 ± 2.20E-02	2.99E-07 ± 1.01E-07	-10.1	-8.9	1.24
BfXusB + ferrichrome	3	2.54E-05	2.46E-04	1.09 ± 1.10E-02	1.76E-07 ± 4.09E-08	-10.3	-9.22	1.11
BfXusB + ferrichrome	4	2.27E-05	2.26E-04	1.06 ± 9.80E-03	2.98E-07 ± 4.67E-08	-12.7	-8.9	3.78

Table S3. Cryo-EM data collection and structure refinement parameters.

	**XusAB**
**Data collection**	
Electron microscope	FEI Titan Krios
Voltage (kV)	300
Spherical aberration (μm)	2.7
Camera	Falcon 4i (counting)
Energy filter	Selectris (10 eV slit)
Magnification	165,000
Pixel size (Å)	0.74
Total dose (e^-/Å^2)	40.83
Defocus range (μm)	-2.0 to -0.8
Number of movies collected	6,645
**Image Processing**	
Symmetry	C1
Initial number of particles	1,110,843
Final number of particles	76,969
Global resolution (FSC = 0.143)	2.7 Å
Map sharpening B-factor (Å^2)	-57.2
**Refinement**	
Model composition	
Non-hydrogen atoms	8,742
Protein residues	1,093
R.m.s. deviations	
Bonds lengths (Å)	0.002
Bond angles (˚)	0.543
Validation	
MolProbity score	1.12
Clash score	2.84
Rotamer outliers (%)	0
Ramachandran plot	
Favoured (%)	97.79
Outliers (%)	0
PDB	9GBC
EMDB	EMD-51210

Table S4. Strains used in this study.

**Strain**	**Description**	**Source**
E. coli TOP10	Used for constructing expression plasmids	Invitrogen
E. coli BL21(DE3)	Used for recombinant protein production	(8)
E. coli S17-1λpir	Used for constructing and conjugating pExchange plasmids into *Bacteroides* spp.	(9)
B. theta VPI-5482 *tdk*$^-$	Thymidine kinase knockout used for allelic exchange with pExchange vector.	(10)
B. theta VPI-5482 *tdk*$^-$ *bt2064-his*	Chromosomal His_6-tag for XusAB complex purification	This study
B. theta VPI-5482 *tdk*$^-$ *bt2064*$^-$ *bt2065*$^-$	*xusAB* knockout	This study
B. fragilis NCTC 9343 *tdk*$^-$	Thymidine kinase knockout used for allelic exchange with pExchange vector.	Janet Quinn (Newcastle)
B. fragilis NCTC 9343 *tdk*$^-$ *bf9343_4228*$^-$ *bf4393_4229*$^-$	*xusAB* knockout	This study

Table S5. Plasmids used in this study.

Strain	Description	Source
pET28b	Used for recombinant protein production	EMD Biosciences
pET28b_BtXusB	Used for expression of BtXusB (residues 35-464)	This study
pET28b_BvXusB	Used for expression of BvXusB (residues 29-491)	This study
pET28b_BfXusB	Used for expression of BfXusB (residues 39-406)	This study
pExchange	Used for *Bacteroides* spp. allelic exchange.	(10)
pExchange_bt2064-his	Used to insert a chromosomal His_6-tag on BtXusB	This study
pExchange_bt2065-64_KO	Used to delete *xusAB* from *B. theta* chromosome	This study
pExchange_bf9343_4229-4228	Used to delete *xusAB* from *B. fragilis* chromosome	This study

Movie S1 (separate file). Morph of the apo BtXusB crystal structure to the FeEnt-bound BtXusB crystal structure. The protein model is depicted as a cartoon and as a surface. The siderophore-binding loops are in blue. The position of FeEnt, shown as an orange stick model, is fixed for reference.

Movie S2 (separate file). Fit of FeEnt and the BtXusB residues and interacting water molecules to the $2mF_o$-DF_c electron density map contoured at 1.5σ. FeEnt is in orange, BtXusB residues are in grey, and water molecules are shown as red spheres.

SI Methods

Growth curve experiments

B. theta tdk⁻ strain was cultured anaerobically in BHI supplemented with hemin at 37 ºC overnight. 0.2 ml of the overnight culture was used to inoculate 5 ml of fresh supplemented BHI the next morning, followed by a 4 h incubation. The cells were collected by centrifugation for 5 min at 2,800 × *g*, 20 ºC, and resuspended in 1 ml of fresh, pre-warmed minimal medium. Minimal medium supplemented with fructose and hemin was aliquoted into tubes, to which BPS, FeEnt and $FeCl_3$ was added as required. Washed cells were diluted in the appropriate tubes to OD_{600}=0.04 and dispensed in 200 µl aliquots into the wells of a sterile 96-well plate in triplicate. Growth at 37 ºC was monitored for 48 h using a Biotek Epoch microplate reader housed inside an anaerobic workstation. The experiment was repeated three times with similar results.

Growth curve experiments

Conditions under which the tagged XusB is expressed were identified by Western blotting. The *B. theta bt_2064-his* strain was cultured anaerobically in tubes containing 2 ml minimal medium supplemented with 6.25-100 μM BPS for 18 h. Equivalents of 1 ml culture at OD_{600}=2 were pelleted by centrifugation. The pellets were resuspended in 80 μl BugBuster (Sigma), supplemented with 1 mM PMSF, and incubated for 15 min at room temperature. Cell debris was pelleted by centrifugation in a benchtop microcentrifuge. Samples from cells grown in the presence of different amounts of BPS were separated by SDS-PAGE and transferred onto a PVDF membrane via wet transfer. The PVDF membrane was stained with Ponceau S stain to confirm successful transfer and blocked with 1% milk solution in PBS supplemented with 0.1% (v/v) Tween 20 for 20 min at room temperature. The membrane was probed with anti-His-horseradish peroxidase conjugate antibody (Roche; 1:500 dilution in 1% milk solution) for 1 h at room temperature and washed three times with PBS-Tween. The blots were developed using SuperSignal West Pico Plus chemiluminescent substrate (Thermo Fisher Scientific) and imaged using a Gel Doc XR+ system (Bio-Rad).

SI References

1. E. Krissinel, K. Henrick, Inference of macromolecular assemblies from crystalline state. *J. Mol. Biol.* **372**, 774–797 (2007).
2. L. Spiga, *et al.*, Iron acquisition by a commensal bacterium modifies host nutritional immunity during *Salmonella* infection. *Cell Host Microbe* **31**, 1639-1654.e10 (2023).

3. A. Punjani, J. L. Rubinstein, D. J. Fleet, M. A. Brubaker, cryoSPARC: algorithms for rapid unsupervised cryo-EM structure determination. *Nat. Methods* **14**, 290–296 (2017).
4. G. Posch, *et al.*, “Cross-glycosylation” of proteins in Bacteroidales species. *Glycobiology* **23**, 568-77 (2013).
5. X. Robert, P. Gouet, Deciphering key features in protein structures with the new ENDscript server. *Nucleic Acids Res.* **42**, W320-W324 (2014).
6. J. Jumper, *et al.*, Highly accurate protein structure prediction with AlphaFold. **596**, 583–589 (2021).
7. N. Oberg, R. Zallot, J. A. Gerlt, EFI-EST, EFI-GNT, and EFI-CGFP: Enzyme Function Initiative (EFI) Web Resource for Genomic Enzymology Tools. *J. Mol. Biol.* **435**, 168018 (2023).
8. F. W. Studier, B. A. Moffatt, Use of bacteriophage T7 RNA polymerase to direct selective high-level expression of cloned genes. *J. Mol. Biol.* **189**, 113–130 (1986).
9. V. de Lorenzo, L. Eltis, B. Kessler, K. N. Timmis, Analysis of *Pseudomonas* gene products using *lacIq/Ptrp-lac* plasmids and transposons that confer conditional phenotypes. *Gene* **123**, 17–24 (1993).
10. N. M. Koropatkin, E. C. Martens, J. I. Gordon, T. J. Smith, Starch Catabolism by a Prominent Human Gut Symbiont Is Directed by the Recognition of Amylose Helices. *Structure* **16**, 1105–1115 (2008).
